# Insights into the mechanism of action of the bipartite fusion module of SARS-CoV-2 spike protein

**DOI:** 10.1128/jvi.00688-26

**Published:** 2026-09-15

**Authors:** Carolina C. Buga, Mariana Valério, Marta Alenquer, Marta Pires de Miranda, Manuel N. Melo, Miguel A. R. B. Castanho, Maria João Amorim, Cláudio M. Soares, João B. Vicente, Ana Salomé Veiga, Diana Lousa

**Affiliations:** 1 Instituto de Tecnologia Química e Biológica António Xavier, Universidade Nova de Lisboa, Oeiras, Portugal; 2 Faculdade de Medicina, Universidade de Lisboa 37811, Lisbon, Portugal; 3 Gulbenkian Institute for Molecular Medicine 705996, Lisbon, Portugal; 4 Católica Biomedical Research Centre, Católica Medical School – Universidade Católica Portuguesa, Oeiras, Portugal; The Ohio State University, Columbus, Ohio, USA

**Keywords:** SARS-CoV-2, viral entry, fusion peptide, membrane fusion, experimental and computational biophysics

## Abstract

SARS-CoV-2 entry into host cells is mediated by the spike glycoprotein, which promotes fusion between viral and host membranes. Despite its importance, the precise location and mode of action of the fusion peptide, a key spike region that inserts into and perturbs the host membrane, remain elusive. Two regions have been proposed as fusion peptides: one located at the N-terminus of the protein (nFP) and the other at an internal position (iFP). Here, we combine computational and experimental approaches to characterize their roles and impact on membrane fusion. Molecular dynamics (MD) simulations indicated that the nFP interacts mostly at the membrane surface. Consistently, experimental biophysical assays revealed that the nFP exhibits low affinity and weak perturbing effects on lipid vesicles. In contrast, the iFP exhibits stronger membrane binding and induces stronger perturbation *in vitro*. MD simulations show that the iFP inserts deeply into and strongly affects the membrane, inducing lipid tail protrusion and increased water flux through the bilayer. Moreover, spike-pseudotyped lentiviruses carrying mutations in the iFP region showed that residues Y873, F888, and F906 are required for viral entry. Together, our findings suggest that SARS-CoV-2 uses a bipartite fusion module in which the nFP establishes initial contact with the host membrane and primes the bilayer, enabling subsequent deep insertion and further membrane destabilization promoted by the iFP. Given that FPs are conserved across viral families, a similar fusion module may be present in other coronaviruses, making this region a promising target for the development of broad-range antiviral therapeutics.

**IMPORTANCE:**

We studied how SARS-CoV-2 enters human cells by focusing on a key region of its spike protein, known as the fusion peptide, which interacts with the host membrane. Although this region is essential for infection, its exact location and mechanism of action have remained unclear, with two candidate regions proposed. Using a combination of computational and experimental approaches, we found that these regions act together through a bipartite fusion module. One region initiates contact with the membrane, while the other inserts more deeply and drives the membrane perturbations required for viral entry. Given the relevance of this peptide for viral entry, these insights are expected to guide the development of novel antiviral therapies.

## INTRODUCTION

**Fig 1:**
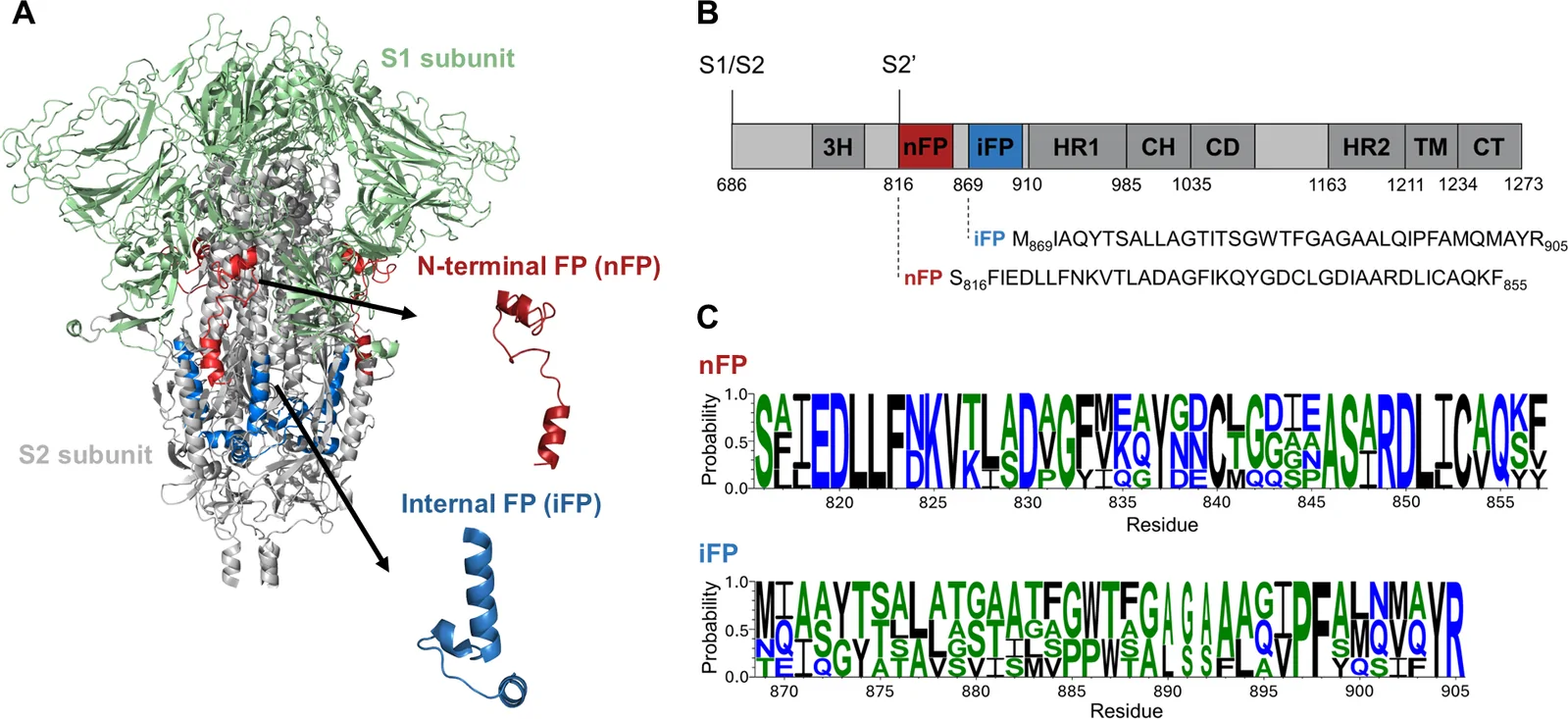
Proposed SARS-CoV-2 FPs. (**A**) Structure of the SARS-CoV-2 spike glycoprotein trimer in its prefusion conformation (model 6VXX_1_1_1 [14]), with proposed FPs highlighted. The S-protein S1 and S2 subunits are shown in green and gray, respectively. The N-terminal fusion peptide (nFP) and the internal fusion peptide (iFP) are shown in red and blue cartoon representation, respectively. (**B**) Schematic representation of SARS-CoV-2 S2 subunit regions including S1/S2 cleavage site (S1/S2), three-helix segment (3H), S2′ cleavage site (S2′), nFP, iFP, heptad repeat 1 (HR1), central helix (CH), connector domain (CD), heptad repeat 2 (HR2), transmembrane anchor (TM), and cytoplasmic tail (CT). nFP and iFP amino acid sequences are displayed below each proposed FP region. (**C**) Sequence conservation of the proposed FPs across representative human coronaviruses (including SARS-CoV-2, SARS-CoV, MERS-CoV, HCoV-OC43, and HCoV-HKU1). Color is used to show hydrophobicity of the residues (blue: hydrophilic, green: neutral, and black: hydrophobic). Sequence logo was generated using WebLogo (15).

The severe acute respiratory syndrome coronavirus 2 (SARS-CoV-2) belongs to the *Betacoronavirus* genus of the *Coronaviridae* family, which also encompasses other viruses relevant for human health such as SARS-CoV, Middle East respiratory syndrome coronavirus (MERS-CoV), human coronavirus OC43 (HCoV-OC43), and human coronavirus HKU1 (HCoV-HKU1). Since the beginning of the 21st century, betacoronaviruses have stood as a global threat to human health and caused significant epidemic outbreaks, including the SARS-CoV outbreak in 2002–2003, the emergence of MERS-CoV in 2012, and, more recently, the COVID-19 pandemic (1–3). These enveloped viruses require fusion between their envelope and the host cell plasma membrane, or the endosomal membrane, for infection to occur. This fusion process is mediated by the spike glycoprotein (S-protein), a class I fusion protein composed of two functional subunits: S1, which is responsible for receptor binding, and S2, which contains the molecular machinery that drives membrane fusion (Fig. 1). Upon receptor binding and proteolytic cleavage, the fusion peptide (FP) is exposed. Viral FPs belonging to class I fusion proteins are usually hydrophobic regions located at the N-terminus of the fusion protein with 20–30 amino acid residues that insert into the target membrane, destabilizing it and initiating the fusion process (4–7). The S-protein trimers then undergo large conformational changes, leading to the formation of a six-helix bundle, promoting membrane fusion through a hemifusion intermediate and, ultimately, adopting the post-fusion conformation (8–11). In contrast, class II (beta-sheet-rich, dimeric) and class III (mixed alpha/beta, trimeric) fusion proteins undergo different structural rearrangements—such as dimer-to-trimer transitions in class II or moderate trimer refolding in class III—and have internal fusion loops rather than N-terminal fusion peptides (12, 13).

The study of viral FPs has gained increasing attention due to their crucial role in viral pathogenesis and their potential as targets for antiviral therapeutics (16–21). SARS-CoV-2 has been no exception, with numerous studies aiming to identify the FP’s location within the S-protein and elucidate its mechanism of action. Two major candidates have been proposed: the N-terminal fusion peptide (nFP, residues 816–855) (22–25) and the internal fusion peptide (iFP, residues 869–905) (22, 26). These proposed FPs are located in close proximity within the S-protein sequence, separated by only 14 amino acid residues. Additionally, it has been proposed that the segments within the region corresponding to the nFP, iFP, and the amino acid residues in between play distinct roles in facilitating membrane fusion (22). In a recent study, we explored various machine learning-based approaches to identify FPs within fusion protein sequences from multiple enveloped viruses, including influenza, HIV, and coronaviruses (27). Interestingly, the models predicted both nFP and iFP as potential FP candidates in coronaviruses, demonstrating that both have sequence patterns compatible with this function.

The nFP and iFP differ not only in their location within the S-protein but also in their sequence conservation among betacoronaviruse*s*, with the nFP being more conserved than the iFP. In addition, they also exhibit different biochemical properties, including amino acid residue composition and hydrophobicity (Fig. 1). The nFP is an amphipathic sequence located at the N-terminus of the S2 subunit, following the S2′ proteolytic cleavage site, and is partially exposed to the solvent in the S-protein prefusion structure. Notably, it has been proposed as an FP in both SARS-CoV and SARS-CoV-2. However, different studies have used slightly different definitions of the nFP region, spanning residues 816–855 (22, 24, 28–35). Most commonly, shorter segments such as amino acid residues 816–833 or 816–841 are studied. Conversely, the iFP is a highly hydrophobic sequence buried within the S2 subunit in the S-protein prefusion structure. Despite the unusual location for a FP belonging to a class I fusion protein, it has been shown to interact with lipid membranes, potentially playing a role in the fusion process. Like the nFP, the iFP’s boundaries are not unanimously defined; its location is generally proposed to be within amino acid residues 869–905 (22, 26, 35–37), with studies focusing on subsets such as 870–888 or 873–894.

Structural and functional approaches have been employed to study the nFP and iFP. Based on nuclear magnetic resonance (NMR) data of the nFP in bilayered micelles, a shallow insertion structure has been proposed. In this structure, the polar region of nFP facilitates attachment by interacting with lipid head groups, but deeper interactions with lipid tails are minimal, likely owing to the presence of charged residues (31). Additionally, deep mutational scanning of a saturation mutagenesis library of the nFP and adjacent residues in authentic SARS-CoV-2, followed by fitness profiling in different cells, showed that the nFP has very low mutational tolerance (24). For the iFP, a cryo-electron microscopy (cryo-EM) structure of post-fusion S-protein in a lipid nanodisc revealed that the peptide is deeply inserted in the membrane, adopting a U-like shape, while part of the nFP region is located in the membrane-solvent interface, and the remaining part is not resolved (26). Moreover, a neutron-scattering study of the SARS-CoV-2 S-protein region corresponding to the nFP, iFP, and the amino acid residues in between revealed that these different regions likely play distinct roles during membrane fusion (22). The data suggest that the nFP appeared to initiate membrane interaction in a calcium-dependent manner, while the iFP contributed to deeper insertion into the lipid bilayer. In SARS-CoV, the regions corresponding to SARS-CoV-2 nFP and iFP have been reported to induce membrane ordering, fusion, and permeabilization (30, 34, 35). Given the high sequence conservation between these regions in SARS-CoV and their counterparts in SARS-CoV-2, it is reasonable to consider that insights gained from studies on SARS-CoV FPs are, at least in part, applicable to the corresponding FPs of SARS-CoV-2.

Despite these advances, significant unanswered questions remain regarding the specific roles of the nFP and iFP in membrane fusion and the molecular mechanism underlying their activity. To address this matter, we combined experimental and computational approaches. Both nFP and iFP were studied using biophysical techniques and lipid vesicles to evaluate their membrane-perturbing effects. Spike-pseudotyped lentiviruses carrying specific amino acid mutations within the SARS-CoV-2 iFP were used to assess the impact of these residues on viral entry. Furthermore, to gain detailed molecular insights, we performed both coarse-grained (CG) and atomistic MD simulations to investigate peptide-membrane and peptide-peptide interactions. We found that although both FPs interact with the membrane, they do it through distinct modes: the nFP inserts more superficially into the membrane, inducing modest membrane perturbations that may prime the bilayer for fusion, whereas the iFP penetrates more deeply into the membrane and exhibits stronger membrane-destabilizing activity. Additionally, mutations within the iFP region abolish pseudovirus infectivity. Based on these findings, we propose that the nFP and iFP act together as a functional fusion module, in which the nFP initiates membrane engagement through an interaction at the membrane surface that facilitates the deeper insertion of the iFP. This sequential interplay enables the iFP to destabilize the membrane, ultimately promoting fusion between the viral envelope and the host cell membrane.

## MATERIALS AND METHODS

### Peptide synthesis

All peptides were synthesized by Bachem AG (Switzerland) using solid-phase peptide synthesis. The nFP comprises the amino acid residues 816–855 (SFIEDLLFNKVTLADAGFIKQYGDCLGDIAARDLICAQKF) of the SARS-CoV-2 B.1 lineage S-protein, while iFP comprises the amino acid residues 869–905 (MIAQYTSALLAGTITSGWTFGAGAALQIPFAMQMAYR) of the same protein. nFP-K and iFP-K derived from nFP and iFP, respectively, were modified with 8-amino-3,6-dioxaoctanoic acid, bridging a tetra-lysine tail at the C-terminus to improve solubility. This strategy has been widely used in viral fusion peptide studies (19, 20, 30, 38–41). The unmodified nFP and iFP peptides retained free C-terminal carboxyl groups. All peptides retained free N-termini, and in all peptides’ syntheses, trifluoroacetic acid was used as the counterion. The molecular weights of the synthesized peptides were determined using mass spectrometry and were 4,424.1 Da for nFP, 3,910.6 Da for iFP, 5,148.96 Da for nFP-K, and 4,635.44 Da for iFP-K. The purity of each peptide was assessed by high-performance liquid chromatography and found to be 88.6% for nFP, 94.5% for iFP, 84.9% for nFP-K, and 89.0% for iFP-K.

### Chemicals and reagents

1-palmitoyl-2-oleoyl-*sn*-glycero-3-phosphocholine (POPC), 1-palmitoyl-2-oleoyl-*sn*-glycero-3-phospho-L-serine (POPS), 1,2-dioleoyl-*sn*-glycero-3-phosphoethanolamine-N-(7-nitro-2-1,3-benzoxadiazol-4-yl) (NBD-PE), and 1,2-dioleoyl-*sn*-glycero-3-phosphoethanolamine-N-(lissamine rhodamine B sulfonyl) (Rhod-PE) were purchased from Avanti Polar Lipids (USA); 5 (6)-carboxyfluorescein (5,6-CF), sodium 2-(4-(2-hydroxyethyl)piperazin-1-yl)ethanesulfonate, 4-(2-hydroxyethyl)piperazine-1-ethanesulfonic acid sodium salt (HEPES), sodium chloride (NaCl), sodium hydroxide (NaOH), sodium dodecyl sulfate (SDS), t-octylphenoxypolyethoxyethanol, and polyethylene glycol tert-octylphenyl ether (Triton X-100) were purchased from Merck (Germany). Dulbecco’s modified Eagle’s medium (DMEM), fetal bovine serum (FBS), penicillin/streptomycin (P/S), glutathione, and phosphate-buffered saline (PBS) were purchased from Thermo Fisher Scientific (USA).

### Lipid vesicle preparation

Large unilamellar vesicles (LUVs) composed of POPC:POPS (4:1 molar ratio) were used as membrane model systems. The lyophilized lipids were solubilized in chloroform in a round-bottom flask, and the organic solvent was then evaporated under a gentle nitrogen flow. The lipidic film was maintained under vacuum overnight. The next day, the lipidic film was rehydrated with 10 mM HEPES, pH 7.4, and 150 mM NaCl and subjected to 10 freeze/thaw cycles. The formed lipidic suspension of multilamellar vesicles was extruded using the LiposoFast-Basic plus Stabilizer setup from Avestin (Germany), with Hamilton (Switzerland) syringes and a 100-nm pore Nucleopore polycarbonate membrane (Whatman/GE Healthcare, United Kingdom).

### Lipid membrane partition

Lipid membrane partition assays were performed by successive additions of POPC:POPS (4:1 molar ratio) LUVs to each peptide solution containing a peptide concentration of 15 μM, covering a range of final lipid concentrations between 0 and 4 mM. After each addition, an incubation for 10 min at 37°C was conducted. The extent of lipid membrane partition was determined following the steady-state fluorescence emission intensity of aromatic amino acid residues, recorded at each lipid concentration. Phenylalanine intrinsic fluorescence was monitored for nFP and nFP-K, using an excitation wavelength of 275 nm (with a 2.5-nm slit) and recording the emission spectra between 285 and 400 nm (with a 3-nm slit). For iFP and iFP-K, tryptophan intrinsic fluorescence was recorded using an excitation wavelength of 290 nm (with a 2.5-nm slit) and recording the emission spectra between 300 and 450 nm (with a 3-nm slit). Each sample was analyzed in three independent experiments in a FLS920 series Edinburgh Instruments spectrofluorometer (United Kingdom). Fluorescence intensity was corrected for dilution, background, and light scattering. The partition coefficient (*K_p_*) value for iFP-K was calculated from a non-linear regression fit of the following equation to experimental data, as described elsewhere (42):


(equation 1) $$\frac{I}{I_{W}}=\;\frac{1+\;K_{p}\gamma_{L}\frac{I_{L}}{I_{W}}\left[L\right]}{1+\;K_{p}\gamma_{L}\left[L\right]}$$

where *I_w_* and *I_L_* are the integrated fluorescence emission intensities in aqueous solution and in lipid, respectively; *γ_L_* is the lipid molar volume; and *[L]* is the lipid concentration.

For nFP and nFP-K, an alternative approach was employed to estimate *K_p_* values, since for these peptides, the relationship of the normalized fluorescence intensity$\;\left(\frac{I}{I_{W}}\right)$ as a function of the lipid concentration ([L]) did not reach close to the limit value, thus making *I_L_/I_w_* impossible to determine, therefore precluding the application of equation 1. At low *K_p_* values and/or high *I_L_/I_w_*, the range of [L] may be too short to leave the initial linear regime of the hyperbola. It is, however, possible to establish a majorant (i.e., upper bound) of *K_p_* from $\frac{d\frac{I}{I_{W}}}{d\left[L\right]}$ at [L] = 0, which we term $m_{0}$, for the sake of simplicity. The first derivative of equation 1 shows that


(equation 2) $$\frac{d\frac{I}{I_{W}}}{d\left[L\right]}\;\left(0\right)\equiv \;m_{0}=\;K_{p}\gamma_{L}\left(\frac{I_{L}}{I_{W}}-1\right)$$

and it can be seen that equating the right-hand side of the equation to an experimentally measured slope leads to an indeterminate system where both *K_p_* and $\frac{I_{L}}{I_{W}}$ are unknowns. equation 2 can be solved for $\frac{I_{L}}{I_{W}}$ :


(equation 3) $$\frac{I_{L}}{I_{W}}\;=\;\frac{m_{0}}{K_{p}\gamma_{L}}+1$$

One can compute the maximum observed $\frac{I}{I_{W}}$ as a proxy of $\frac{I_{L}}{I_{W}}$, thus being an apparent maximum value we name ${\left(\frac{I_{L}}{I_{W}}\right)}_{app}$. The apparent value is an underestimate of the real value:


(equation 4) $${\left(\frac{I_{L}}{I_{W}}\right)}_{app}\leq \frac{\;I_{L}}{I_{W}}$$

which, combined with equation 3, yields:


(equation 5) $${\left(\frac{I_{L}}{I_{W}}\right)}_{app}\leq \frac{m_{0}}{K_{p}\gamma_{L}}+1$$

solving for *K_p_* :


(equation 6) $$K_{p}\leq \frac{m_{0}}{\gamma_{L}\left[{\left(\frac{I_{L}}{I_{W}}\right)}_{app}-1\right]\;}$$

Now, when data points are close to linear, one can approximate $m_{0}$ from the slope of the segment between the first and last point of the curve (where the largest lipid concentration used is termed ${\left[L\right]}_{max}$):


(equation 7) $$m_{0}\approx \frac{\frac{I}{I_{W}}\;\left({\left[L\right]}_{max}\right)\;-\;1}{{\left[L\right]}_{max}}$$

Because $\frac{I}{I_{W}}\left({\left[L\right]}_{max}\right)$ is the largest observed value for $\frac{I}{I_{W}}$ :


(equation 8) $$\frac{I}{I_{W}}\left({\left[L\right]}_{max}\right)={\left(\frac{I_{L}}{I_{W}}\right)}_{app}$$

Combining equation 7 and equation 8, this yields:


(equation 9) $$m_{0}\approx \frac{{\left(\frac{I_{L}}{I_{W}}\right)}_{app}-\;1}{{\left[L\right]}_{max}}$$

Plugging it into equation 6:


(equation 10) $$\begin{array}{c} K_{p}\leq \frac{\frac{{\left(I_{L}/I_{W}\right)}_{app}-1}{[L{]}_{\max}}}{\gamma_{L}\left[{\left(I_{L}/I_{W}\right)}_{app}-1\right]}\Leftrightarrow \\ K_{p}\leq \frac{1}{\gamma_{L}[L{]}_{\max}} \end{array}$$

equation 10 allows an estimation of the upper bounds of *K_p_* from experimental data.

### Lipid mixing

A Förster resonance energy transfer (FRET) approach was used to evaluate the lipid mixing induced by the peptides. POPC:POPS (4:1 molar ratio) LUVs labeled with both 1% N-NBD-PE (donor) and 1% N-Rh-PE (acceptor) and unlabeled vesicles were mixed in a 1:4 ratio in 10 mM HEPES, pH 7.4, and 150 mM NaCl. A peptide-to-lipid molar ratio of 0.15 was tested for each peptide in three independent experiments. The fluorescence emission spectrum of each sample was measured in a FLS920 series Edinburgh Instruments spectrofluorometer between 500 and 650 nm (with a 10-nm slit) with an excitation wavelength of 470 nm (with a 3-nm slit). The fusion efficiency (*R*) was calculated as follows:


(equation 11) $$R=\frac{IF_{530nm}}{IF_{588nm}}$$

where IF_530nm_ and IF_588nm_ correspond to the fluorescence emission intensity recorded at 530 nm and 588 nm, respectively. The percentage of lipid mixing was then determined using the following equation:


(equation 12) $$Lipid\;mixing\left(\%\right)=\frac{R_{S}-R_{0}}{R_{max}-R_{0}}\times 100$$

where *R_S_* is the sample fusion efficiency, *R*_0_ is the fusion efficiency of the negative control (a vesicle mixture in the absence of peptide), and *R*_max_ is the fusion efficiency for the positive control (a vesicle mixture in the presence of 1% [vol/vol] Triton X-100).

### Lipid vesicle content leakage

The peptide-induced vesicle content leakage of POPC:POPS (4:1) LUVs loaded with 50 mM 5,6-carboxyfluorescein (5,6-CF) was determined by the dequenching of 5,6-CF upon release from the vesicles, as described elsewhere (43). Briefly, each peptide was incubated in 10 mM HEPES, pH 7.4, and 150 mM NaCl with LUVs in peptide-to-lipid molar ratios ranging from 0 to 0.25 for 15 min at 37°C. The fluorescence emission intensity was measured at 520 nm in a Varian Cary Eclipse spectrofluorometer (United Kingdom), with an excitation wavelength of 493 nm. The percentage of vesicle content leakage induced by each peptide was determined in three independent experiments, as follows:


(equation 13) $$Leakage\left(\%\right)=\frac{F_{S}-F_{0}}{F_{max}-F_{0}}\times 100$$

where *F_S_* is the fluorescence emission intensity measured after the 15-min incubation of each peptide with the LUVs, *F*_0_ is the negative control, corresponding to the fluorescence emission intensity of the LUVs measured in the absence of peptide, and *F*_max_ is the positive control, corresponding to the fluorescence emission intensity measured after the addition of 1% (vol/vol) Triton X-100.

### Lipid vesicle aggregation

The aggregation of lipid vesicles promoted by the peptides was determined by dynamic light scattering (DLS). Titration of lipid vesicles with peptide up to peptide-to-lipid molar ratios between 0 and 0.25 was performed using POPC:POPS (4:1 molar ratio) LUVs in 10 mM HEPES, pH 7.4, and 150 mM NaCl. After each addition, an incubation of 5 min at 37°C was conducted, and samples were analyzed in a Malvern Instruments ZetasizerNano ZS (United Kingdom). Three independent experiments were performed, each corresponding to an average autocorrelation curve obtained from at least 10 replicate sample scans. The CONTIN method (44) was applied to determine the mean diffusion coefficient (*D*) values of the vesicles of each sample. *D* values were then used to calculate the particles’ Z-average hydrodynamic diameter through the Stokes-Einstein-Sutherland equation (45):


(equation 14) $$D=\;\frac{k_{B}T}{6\pi \eta r}$$

where *k_B_* is the Boltzmann constant (1.3807 × 10⁻²³ J/K), *T* is the absolute temperature (K), η is the dynamic viscosity of the solvent (Pa·s), and *r* is the hydrodynamic radius (m).

### Pseudovirus production and characterization

Pseudoviruses were produced by transfecting HEK293ET cells. Cells were seeded 24 h prior to transfection at a density of 4 × 10⁶ cells per 10-cm dish in DMEM supplemented with 10% (vol/vol) FBS, 1% (vol/vol) P/S, and 1% (vol/vol) glutathione. On the day of transfection, the cells were co-transfected using a calcium phosphate transfection method, as described elsewhere (46), with the following plasmids: pLEX-GFP, generated in-house by subcloning GFP into the pLEX.MCS backbone; psPAX2, which was a kind gift from Dr. Luís Moita (Faculdade de Medicina, Universidade de Lisboa, Portugal); and pCAGGS-spike encoding SARS-CoV-2 S-protein sequences with or without mutations, custom-synthesized by GeneCust (France). At 6 h post-transfection, the cells were washed with PBS, and fresh culture medium was added. Cells were incubated at 37°C with 5% CO₂. At 3 days post-transfection, the culture supernatant containing pseudoviruses was harvested, clarified by low-speed centrifugation, and concentrated using the Lenti-X Concentrator (Takara Bio), following the manufacturer’s protocol. Pseudovirus stocks were aliquoted and stored at –80°C until use. Following production, pseudovirus stocks were characterized to assess both the quantity of lentiviral particles and the presence of the SARS-CoV-2 S-protein. The total number of lentiviral particles was determined using the Lenti-X p24 Rapid Titer Kit (Takara Bio, USA). To check for S-protein incorporation, western blot analysis was first performed using the polyclonal antibody #PA5-81795 (Thermo Fisher, USA) against the S-protein S1 subunit. Additionally, the amount of S-protein incorporated into the pseudoviruses was quantified using the SARS-CoV-2 Spike Protein ELISA Kit (AcroBiosystems, USA). Three independent pseudovirus productions were made.

### Pseudovirus binding to ACE2 on cell surface

To assess the binding capacity of spike-pseudotyped lentiviral particles to the receptor ACE2, HEK293T cells stably expressing human ACE2 (HEK293T-ACE2) and HEK293T cells (negative control) were used; 2 × 10^5^ cells of each cell line were incubated with 7.43 × 10^8^ pseudoviral particles in PBS supplemented with 2% FBS for 1 h at 4°C. This temperature was used to allow receptor-mediated attachment while preventing membrane fusion and endocytosis. Following incubation, cells were centrifuged at 300 × *g* for 3 min at 4°C, and supernatants were discarded. Cells were washed three times with 1 mL PBS containing 2% FBS, with centrifugation between washes. The final cell pellet was resuspended in 200 µL PBS with 2% FBS and transferred to 96-well V-bottom plates. Bound pseudoviruses were detected by incubation with the polyclonal antibody #PA5-81795 (Thermo Fisher, USA) for 1 h at 4°C. After three washes with 200 µL PBS and 2% FBS, the cells were incubated with the Alexa Fluor 488-conjugated anti-rabbit secondary antibody #A-21206 (Thermo Fisher, USA) for 20 min at 4°C. Cells were washed twice, fixed with 3% paraformaldehyde for 15 min at room temperature, and analyzed by flow cytometry using a BD LSRFortessa SORP (BD). FlowJo software (BD) was used to determine the percentage of cells positive for the Alexa Fluor 488-conjugated antibody, reflecting pseudovirus binding to ACE2 on the cell surface. Two independent experiments were performed.

### Infection assay

To assess pseudovirus infectivity, a titration assay was performed using HEK293T-ACE2. Cells were seeded at a density of 5 × 10⁴ cells per well in a 24-well plate 24 h before infection, using DMEM supplemented with 10% (vol/vol) FBS, 1% (vol/vol) P/S, and 1% (vol/vol) glutathione. On the day of infection, pseudovirus stocks were serially diluted (3-fold dilutions), and 250 µL of each dilution were added to the cells. Infections were carried out at 37°C with 5% CO₂. After a 4-h incubation, the medium was replaced with a fresh growth medium, DMEM supplemented with 10% (vol/vol) FBS, 1% (vol/vol) P/S, and 1% (vol/vol) glutathione. At 24 h post-infection, cells were harvested and analyzed in a Becton Dickinson (BD) LSR Fortessa X-20 equipped with FACS Diva and FlowJo (BD) software to determine the percentage of GFP-positive cells, which reflects the infection efficiency of each pseudovirus. Three independent experiments for each pseudovirus were performed.

### System preparation for MD simulations

The systems modeled in MD simulations for each of the proposed SARS-CoV-2 FP were constructed using different strategies, reflecting the availability of experimental structural data at the time the study began. For the nFP, an experimentally resolved NMR structure in a lipidic environment was available (PDB ID: 7MY8). This structure, solved in bilayered micelles, revealed a wedge-shaped conformation composed of three helices: helices 1 and 2 interact with the hydrophobic lipid tails, while helix 3 remains partially exposed to the aqueous environment. In contrast, no lipid-bound structure was initially available for the iFP. To explore its possible membrane-binding configurations, we designed two systems: a lying configuration, where the peptide rests atop the membrane surface, and an inserted configuration, where the peptide is pre-inserted into the bilayer. However, a recently published cryo-EM structure of the post-fusion SARS-CoV-2 spike glycoprotein embedded in a lipid nanodisc confirmed the insertion of iFP into the membrane (26), validating our inserted setup. Therefore, all analyses in the present study focus on the iFP-inserted system.

### Coarse-grain MD simulations

Since the organization of multiple peptide copies within the membrane is not known *a priori*, CG simulations were employed to efficiently explore the most probable arrangements while keeping computational resources manageable. We used the NMR structure of nFP in a lipidic environment (PDB ID: 7MY8) as the starting conformation and converted it to a Martini coarse-grained topology (47, 48) with the martinize2 program (49). This program was also used to generate the topology and structure files based on the atomistic PDB file. Triplicate coarse-grained (CG) simulations of both the nFP and iFP systems were performed in a POPC:POPS (4:1 molar ratio) membrane to closely mimic the experimental conditions. Consistent with previous studies (19, 20), spontaneous peptide insertion was not simulated. Instead, the peptides were initialized in a pre-inserted membrane-bound configuration, since the extensive conformational sampling required to capture membrane insertion events accurately would imply a prohibitive computational cost. The scope of this analysis was not to study the membrane insertion process, but rather to provide molecular insights into the molecular mechanisms by which the peptides interact with and destabilize the membrane once they are inserted. To mimic conditions of high peptide concentrations, each system was set up with approximately one peptide for every 80 lipids, resulting in an average of 8 peptides and 640 lipids per simulation box. The N-termini of the peptides were considered protonated, and the C-termini were considered amidated.

The system was built using the INSANE script (50), in a 15 × 15 × 12 nm box. The total charge of the systems was first neutralized with either Na^+^ or Cl^−^ ions, and an additional 140 mM NaCl was then added. All simulations were performed using GROMACS 2020.3 package (51), using the Martini 3 force field (48) and lipid topologies released with it. The systems were energy-minimized using the steepest-descent method for 5,000 steps. In this step, the peptides’ backbone atoms were position-restrained using a force constant of 500 kJ/mol/nm^2^. The simulations were run in the isothermal-isobaric (NPT) ensemble, coupling the system to a temperature and pressure bath using the v-rescale and Parrinello-Rahman (52) schemes, respectively. Before performing production runs, an equilibration step was carried out without any restraints for 20 ns, using the v-rescale thermostat and Berendsen barostat to equilibrate temperature and pressure. The target temperature was set to 310 K using two coupling groups: one containing the peptides and membrane, and the other containing the solvent. Semi-isotropic pressure coupling was set to a target pressure of 1 atm using a compressibility of 3 × 10^−4^ bar^−1^. Reaction-field electrostatics were employed, and Lennard-Jones and Coulombic interactions were both cut off at 1.1 nm (53). Each system was run for 30 µs.

### Atomistic MD simulations

Atomistic MD simulations of the iFP and nFP were performed using the last frame of the CG simulations as the starting structure. The systems were converted from coarse-grain to atomistic detail using the backward.py script (54). All atomistic simulations were performed using the GROMACS 2020.3 (51) package and the CHARMM36m force field (55). The system was energy-minimized for 5,000 steps using the steepest-descent algorithm. Following energy minimization, the system was equilibrated. The temperature was maintained at 310 K using a v-rescale thermostat (56), with a coupling constant of 1 ps. Two temperature-coupling groups were defined: one containing the peptides and membrane, and the other containing the solvent. Pressure was controlled using the C-rescale barostat with semi-isotropic coupling and a coupling constant of 5 ps (57). During the equilibration, atomic position restraints on the protein heavy atoms were gradually relaxed in six steps from 4,000 to 2,000 kJ/mol/nm^2^ and from 500 to 200 kJ/mol/nm^2^ on the protein backbone and side chains, respectively. The lipid’s phosphate atoms were also gradually relaxed from 1,000 to 40 kJ/mol/nm^2^. After the sixth step, production simulations were performed.

Simulations were run in the isothermal-isobaric (NPT) ensemble, coupling the system to a temperature and pressure bath using the v-rescale (56) and C-rescale (57) schemes, respectively. The target temperature was set to 310 K, again using two coupling groups, with a coupling constant of 1 ps. Semi-isotropic pressure coupling was set to 1 atm, with a coupling constant of 5 ps and using a compressibility of 4.5 × 10^−5^ bar^−1^. Long-range electrostatic interactions were treated with the Particle Mesh Ewald (PME) (58) scheme, using a grid spacing of 0.12 nm, with cubic interpolation. The neighbor list was updated every 20 steps using a Verlet cutoff with a 1.2-nm radius. Rigid bonds involving hydrogen atoms were constrained using the LINear Constraint Solver (LINCS) algorithm (59). Three replicates were simulated for each system, with all simulations being run for 1 µs.

### MD simulation analysis tools

Atomistic MD simulations were analyzed using 2,500 frames sampled from the last 500 µs of each replicate. Membrane thickness was determined from the z-coordinates of phosphate atoms: kernel density estimation was applied to the z-distributions to identify the peak positions corresponding to the upper and lower membrane leaflets, and thickness was calculated as the distance between these peaks. To evaluate FP-induced membrane perturbations, lipid tail protrusion events were quantified using a custom Python script that counts lipids with atoms within 4 Å of the peptides and in which at least one tail carbon atom extended out of the bilayer plane more than 0.1 nm beyond the phosphate headgroup (19, 20). Water flux was assessed by combining the HOLE method (60, 61) to calculate the space available within the membrane for water molecules with the fluxer.py script (62) to count water molecules crossing the membrane. Interpeptide interactions were quantified by measuring interatomic distances, with contacts defined as atoms within 5 Å. Finally, representative snapshots were visualized and rendered in VMD (63).

## RESULTS AND DISCUSSION

In this work, we studied the interaction with membranes and the effect on membrane properties of two regions of the SARS-CoV-2 S-protein that have been proposed as FPs: the nFP (residues 816–855) and the iFP (residues 869–905). While previous studies focused on different, partially overlapping segments within these regions, we analyzed the full sequences most commonly described in the literature for nFP and iFP. Using the extended peptide regions ensured that we did not overlook amino acid residues relevant to the function of the FP segments. The peptides were studied in their original amino acid residue sequence and with a poly-lysine C-terminal tail to improve solubility (named nFP-K and iFP-K). The addition of a poly-lysine tail might alter the biochemical properties of the peptides and therefore represents an inherent limitation of the studies. However, it is required to improve the solubility of the fusion peptides, facilitating experimental studies.

### nFP and iFP exhibit different membrane affinity

**Fig 2:**
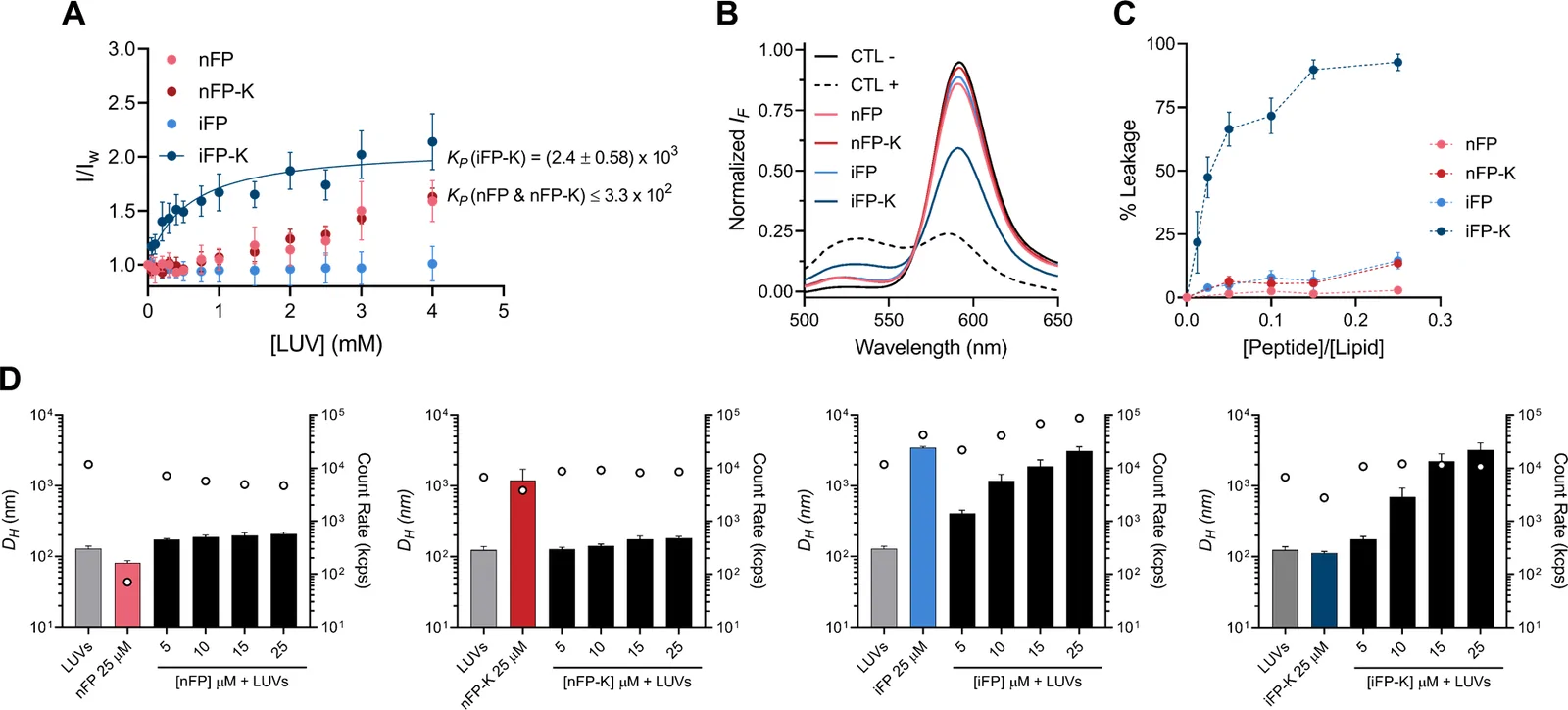
Characterization of proposed SARS-CoV-2 FPs *in vitro*. (**A**) Membrane partitioning of FPs. The ratio of fluorescence emission intensity in the presence of LUVs (*I*) to that in aqueous solution (*I_W_*) was plotted as a function of LUV concentration. Equation 1 was used to fit iFP-K data and determine the partition coefficient (*K_P_*), whereas equation 10 was used to estimate the upper bounds of nFP and nFP-K *K_p_*. (**B**) Representative FRET spectra of FP-induced lipid mixing in POPC:POPS (4:1 molar ratio) LUVs. Solid black line represents the negative control, while dashed black line corresponds to the positive control (1% Triton X-100, vol/vol). (**C**) Percentage of FPs-induced lipid vesicle content leakage at an increasing ratio of peptide-to-lipid concentrations. (**D**) FPs and FPs-induced lipid vesicle aggregation profiles. Monitoring with dynamic light scattering of Z-average hydrodynamic diameter (*D_H_*, columns) and count rate (circles) of particles formed during FPs titration in LUV solutions. Aggregation profiles of LUVs and FPs alone were used as controls. In all panels, nFP is shown in pink, nFP-K in red, iFP in light blue, and iFP-K in dark blue. Error bars indicate the standard deviation from three independent experiments.

As the fusion of the viral envelope with host membranes requires an intimate interaction between these lipids and the S-protein, and given the different biochemical properties of the proposed SARS-CoV-2 FPs, we first assessed the lipid membrane-binding affinities of nFP and iFP. For this, we used LUVs composed of POPC:POPS and employed steady-state fluorescence spectroscopy, taking advantage of the optical sensitivity of the aromatic amino acid residues of the peptides to their local environment. Membrane partition was quantified by monitoring changes in the peptide fluorescence emission intensity upon successive additions of POPC:POPS (4:1 molar ratio) LUVs. The partition coefficient (*K*_*p*_) for iFP-K was determined by plotting normalized fluorescence emission intensity as a function of lipid concentration and using equation 1, whereas for nFP and nFP-K, a maximum *K*_*p*_ value was estimated using Eq. 10 (Fig. 2A). iFP-K exhibits the highest membrane affinity, with a *K_p_* of (2.4 ± 0.58) × 10^3^. nFP and nFP-K have maximum *K_p_* values of 3.3 × 10^2^. The iFP either binds membranes very weakly or undergoes no fluorescence emission change upon binding; in either case, *K_p_* fitting was not possible.

The addition of the poly-lysine tail to nFP did not significantly change its membrane partitioning behavior, probably due to its already amphipathic nature. In contrast, the different membrane partition observed for iFP-K compared to iFP likely arises from changes in the physicochemical properties of the peptide introduced by the poly-lysine tail, including reduced hydrophobic self-association in aqueous solution and possible effects on Trp fluorescence in different environments. While the cationic poly-lysine tail may enhance membrane binding through electrostatic interactions with the anionic groups of membrane phospholipids, these alone cannot account for the increased membrane affinity, as similar effects would be expected between nFP and nFP-K. Indeed, we observe that the high hydrophobicity of iFP leads to self-aggregation in aqueous solution, which is decreased by the poly-lysine tail (Fig. 2D). Improving solubility also likely increases availability for interaction with the lipid membranes. It is also worth noting that we observed lower fluorescence intensity for iFP-K than for iFP in aqueous solution under the conditions used for the lipid membrane partition assays, which may reflect partial quenching of the Trp residue by the poly-lysine tail. Upon membrane insertion, where the Trp becomes more shielded from the aqueous environment, this quenching would be reduced, potentially contributing to the larger relative fluorescence increase observed for iFP-K. In the S-protein, iFP is framed by its flanking sequences, which likely limit both peptide self-aggregation and solvent exposure of the Trp residue. In this sense, iFP-K is a better model of the S-protein-inserted iFP than free iFP itself and is in line with cryo-EM structures of the post-fusion spike protein (26) where the iFP inserts into lipid nanodiscs.

### iFP promotes lipid mixing and lipid vesicle content leakage

To better understand the role of nFP and iFP in the membrane fusion process, their membrane-perturbing properties were evaluated using fluorescence-based assays. Lipid mixing was assessed using a FRET-based approach, while lipid vesicle content leakage was assessed through the release of 5,6-CF. iFP-K had the most pronounced effect in both assays inducing 15% lipid mixing (Fig. 2B) and exhibiting a concentration-dependent increase in lipid vesicle content leakage, reaching 90% at the highest peptide-to-lipid ratio (Fig. 2C). In contrast, nFP, nFP-K, and iFP showed minimal lipid mixing (~2%, Fig. 2B). Lipid vesicle content leakage was also low for these peptides: iFP and nFP-K induced modest leakage of 15% at the highest peptide-to-lipid ratio tested, while nFP had no detectable effect (Fig. 2C). Collectively, the results show that the effects of all peptides on membranes are consistent between assays. The enhanced membrane-perturbing effects of iFP-K when compared with iFP can be assigned to the presence of the poly-lysine tail, which improves solubility and, in turn, impacts the ability to interact with lipids, as previously discussed.

### iFP induces lipid vesicle aggregation

Oligomerization or aggregation of viral FPs on the cell membrane surface is a characteristic shared by many viruses (19, 64–67). *In vitro*, the accumulation of peptides on lipid vesicle surfaces may promote the destabilization of lipid-lipid interactions and affect local membrane properties, such as curvature and tension, which, under certain conditions, can lead to vesicle clustering or aggregation (19, 67). To determine whether any of the proposed SARS-CoV-2 FPs exhibit this behavior, we monitored vesicle aggregation induced by the peptides using DLS by measuring the mean hydrodynamic diameter (*D_H_*) and particle count rate (Fig. 2D). On the one hand, neither nFP nor nFP-K induced extensive vesicle aggregation. Interestingly, nFP-K forms significantly larger aggregates than nFP (Fig. 2D), showing self-association with the formation of micrometer-sized clusters in aqueous solution. In contrast, nFP particles in suspension have a smaller size, but the severe decrease in scattered light intensity shows that part of the peptide precipitates. Interestingly, both peptides exhibit identical membrane partition constants and membrane-perturbing effects, indicating that lipid-peptide interactions override peptide-peptide interactions. This is consistent with the amphipathic nature of these peptides.

Both iFP and iFP-K affected vesicle size distributions, causing a concentration-dependent increase in vesicles’ aggregation, i.e., *D_H_*. iFP forms large micrometer-sized aggregates in solution in the absence of vesicles—even larger than nFP’s—in contrast with the small size determined for iFP-K and consistent with the improved solubility of the latter. Interestingly, DLS shows that iFP interacts with vesicles despite the weak binding profile observed by fluorescence. It is possible that iFP clusters vesicles while maintaining its self-aggregation status. This would keep the aromatic residue fluorophores shielded from contact with lipids and yield an apparent low *K_p_*.

Although both nFP and nFP-K interact with the membrane, neither peptide induces significant lipid mixing, lipid vesicle content leakage, or lipid vesicle aggregation. This suggests that membrane binding alone is not sufficient to induce membrane perturbation. In contrast, iFP-K exhibited the highest membrane affinity and perturbation effects among all tested peptides, highlighting its potential relevance in the context of viral membrane fusion. Based on these results, and given that deep mutational scanning has already been conducted for the nFP (24), we focused our subsequent analysis on the iFP. Within this peptide, we specifically targeted aromatic residues, as they are known to play a key role in membrane interaction and perturbation and are highly conserved across coronaviruses (Fig. 1C), suggesting a functional role in viral entry.

### iFP F898 is essential for S-protein incorporation in pseudoviral particles

To evaluate the impact of SARS-CoV-2 iFP aromatic residues on viral entry, we assessed the infectivity of spike-pseudotyped lentiviruses carrying specific mutations in positions corresponding to these residues (Table S1). We used a spike-pseudotyped lentiviral system bearing the SARS-CoV-2 S-protein from the D614G variant (B.1 lineage), which became globally dominant from mid-2020 and is widely used as a reference in experimental studies (68–73). In our experiments, this variant was considered the wild type (WT), and all other mutant pseudoviruses were compared against this WT reference.

While we focused essentially on the iFP region spanning amino acid residues 869–905 of the S-protein, a mutation on F906 was also included since this residue could contribute to the peptide’s membrane interactions and membrane perturbation effects. When mutating, two main types of amino acid residue replacements were selected: alanine and aromatic residues differing from those in the native sequence. Alanine was chosen because it removes the side chain beyond the beta-carbon with minimal steric or electrostatic effects, allowing the assessment of side chain contributions while preserving the overall peptide structure (74). Replacement of each aromatic residue with other aromatic residues allowed us to evaluate whether their function depends on specific side chain interactions or if a general aromatic character is sufficient to maintain infectivity. The SARS-CoV-2 S-protein is displayed on the surface of pseudoviruses, enabling entry into host cells via the same mechanism as the authentic virus. In addition, a GFP reporter system was included to allow the quantification of infected cells. Although these pseudoviruses mimic the entry process of SARS-CoV-2, they are non-replicative and do not contain the pathogenic viral genome. As such, they are limited to a single-cycle infection: they can enter host cells and deliver their genetic material, but they cannot produce new infectious particles. Following entry, the host cell machinery expresses the GFP reporter, enabling measurement of infection efficiency without further viral replication. Therefore, the only step of the viral infection cycle being analyzed is viral entry.

To confirm S-protein incorporation into the pseudoviruses, western blot analysis was performed on viral particle suspensions. Most pseudovirus mutants successfully incorporated the S-protein, whereas pseudoviruses carrying F898 mutations lacked detectable S-protein (Fig. S1), suggesting that F898 might play a key structural role in maintaining S-protein stability or its incorporation. With S-protein incorporation confirmed for all other mutants, we proceeded to assess the functional consequences of the remaining aromatic residue mutations on viral entry.

### iFP Y873, F888, and F906 are essential for viral entry

**Fig 3:**
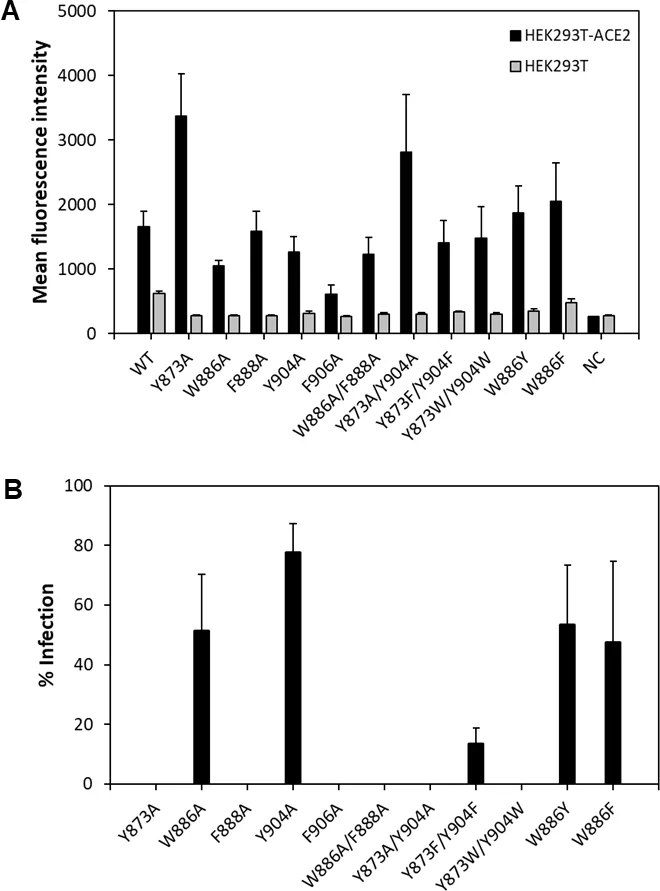
(**A**) Binding of S-protein mutant pseudoviruses to HEK293T-ACE2 and HEK293T cells was assessed by flow cytometry using a polyclonal anti-S1 antibody conjugated to Alexa Fluor 488. Data are presented as mean fluorescence intensity from two independent experiments, and error bars indicate standard deviation. (**B**) Infectivity of S-protein mutant pseudoviruses relative to SARS-CoV-2 WT reference. The infectivity of lentiviral pseudoviruses bearing various S-protein mutations was assessed relative to a pseudovirus displaying the SARS-CoV-2 D614G S-protein (B.1 lineage). Viral entry efficiency was measured in HEK293T-ACE2 target cells by quantifying the proportion of GFP-positive cells using flow cytometry. Data represent mean values from three independent experiments, and error bars represent the corresponding standard deviation.

To assess whether the S-protein present on the pseudoviruses retained the correct prefusion conformation, their ability to bind to the receptor ACE2 was evaluated. As shown in Fig. 3A, the 11 pseudoviral particles with detectable S-protein exhibited measurable binding to ACE2 expressed on the surface of HEK293T-ACE2 cells, while no binding was observed to control HEK293T cells lacking ACE2. The pseudoviral particles carrying S-protein mutations exhibited different levels of ACE2 binding, with the F906A mutant showing the lowest binding capacity compared with the WT pseudovirus. Despite these differences, the results suggest that the incorporated S-proteins retained a correctly folded prefusion conformation capable of binding ACE2, indicating that the introduced mutations did not abolish receptor binding.

To evaluate the infectivity of pseudoviruses displaying the S-protein, an infectivity assay was performed in HEK293T-ACE2 cells. Infection efficiency was quantified by measuring the percentage of GFP-positive cells via flow cytometry, providing a direct readout of viral entry mediated by each S-protein mutant (Fig. 3B). Pseudoviruses carrying mutations at residues F888 or F906 completely lost their ability to mediate infection, indicating that these residues play a vital functional role during viral entry, possibly being involved in maintaining interactions necessary for membrane fusion. Interestingly, the Y873A mutation also abolished viral infectivity, highlighting the importance of this residue. However, when Y873 was replaced by phenylalanine (Y873F), the pseudoviruses partially retained their infectivity. Notably, this mutation was tested in combination with Y904F, generating a Y873F/Y904F double mutant that retained detectable entry. This supports the idea that aromaticity at this position is essential. Mutation at Y904 to alanine (Y904A) resulted in a moderate reduction in infectivity but did not abolish viral entry, suggesting that while Y904 contributes to optimal S-protein function during entry, it is not absolutely required. W886 mutations to either alanine or another aromatic residue resulted in a nearly 50% loss of infectivity. Given that all pseudotyped viruses retained the ability to bind to ACE2, the effect of the mutations is due to their impact on other steps of the entry process, supporting the hypothesis that the iFP has a key role in membrane fusion.

While the infectivity assays highlighted the functional importance of specific aromatic residues within the iFP, they did not directly reveal how these residues contribute to membrane interaction or fusion at the molecular level. MD simulations were the strategy selected to gain deeper insight into the molecular mechanisms underlying the interactions of both the iFP and the nFP with the membrane.

### CG MD simulations predict that the iFP and iFP-K form oligomeric assemblies in the membrane

To compare and validate our experimental findings, we performed MD simulations of multiple copies of nFP, nFP-K, iFP, and iFP-K embedded in POPC:POPS lipid bilayers. The simulations were initialized with the peptides pre-inserted into the membrane, as explained in the methods section. This strategy was tested and validated in previous studies (19, 20). Simulating spontaneous insertion from solution was avoided due to the significant computational cost associated with sampling the extensive conformational space required for accurate insertion events. While experimental data indicate the differences in membrane partitioning among the peptides, our simulations were designed to investigate and compare their behavior once already inserted into the membrane. The primary goal was to gain mechanistic insights into how each of the proposed FPs interacts with and perturbs the membrane.

**Fig 4:**
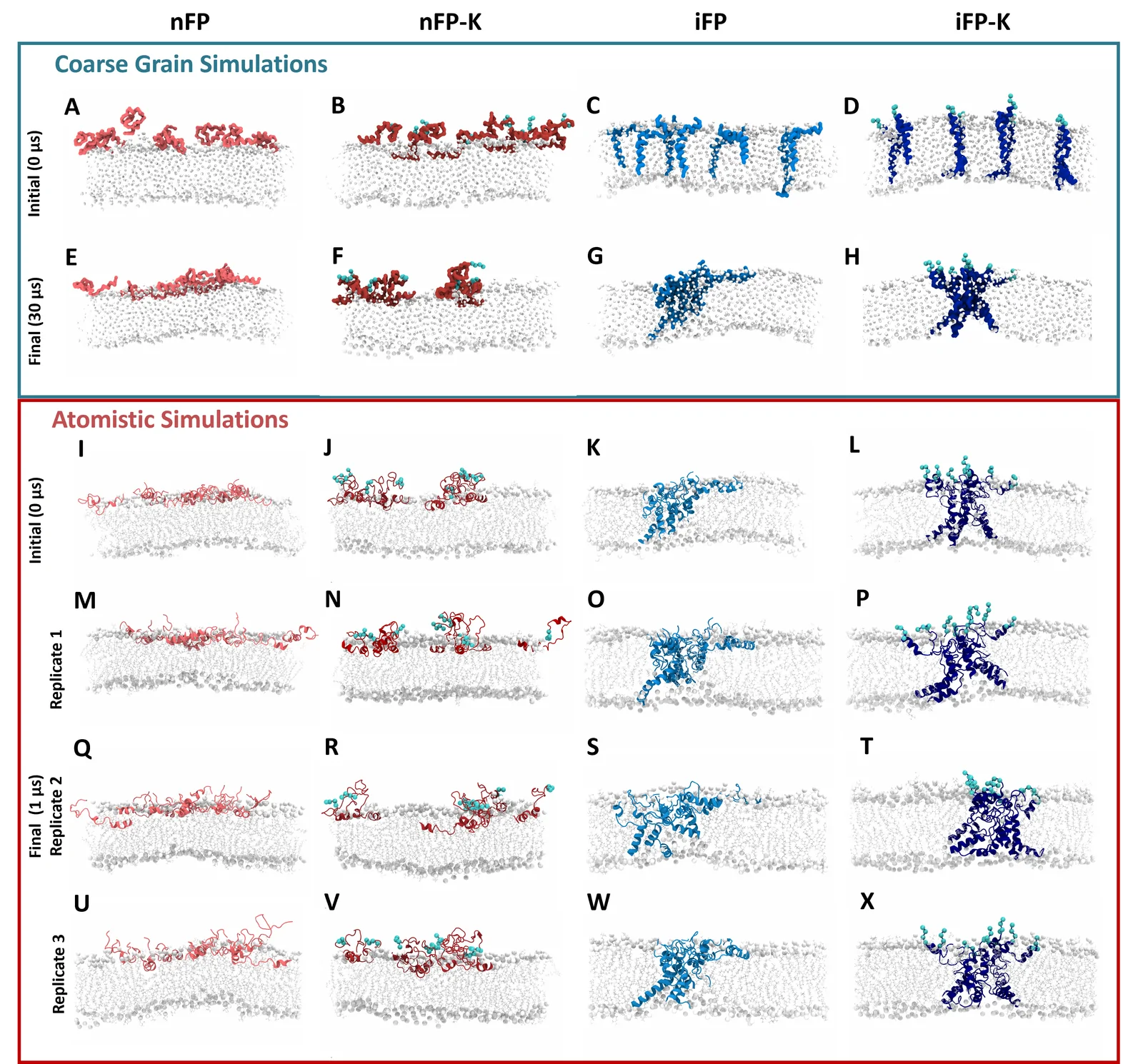
Snapshots of the initial and final frames of the CG and atomistic MD simulations. Initially, eight copies of the proposed SARS-CoV-2 FPs were either placed in POPC:POPS (4:1 molar ratio) membranes (**A–D**). The four systems were then simulated at CG resolution for 30 μs (**E–H**). The last frame of the CG simulation was converted to atomistic detail using the backward tool (54) (**I–L**). Three independent replicates of each system were then simulated for 1 μs (nFP: **M, Q, and U**; nFP-K: **N, R, and V**; iFP: **O, S, and W**; and iFP-K: **P, T, and X**). Lipids are shown in white, while the peptides iFP, iFP-K, nFP, and nFP-K are shown in pink, red, light blue, and dark blue, respectively. The backbone of the lysine tail amino acids is highlighted in cyan.

During CG simulations, nFP and nFP-K peptides were observed to aggregate within the membrane, forming clusters composed of three to four peptide copies (Fig. 4E and F). Similar aggregation was observed in the iFP and iFP-K systems; however, in these cases, all peptide copies in the simulation system contributed to the formation of larger oligomeric assemblies. These assemblies adopted a transmembrane bundle-like conformation (Fig. 4G and H). A similar aggregation mechanism within the membrane has been observed for other fusion peptides from class I fusion proteins, suggesting a common mechanism for promoting membrane fusion (19, 20). The resulting structures from the CG simulations were subsequently used as initial conformations for atomistic MD simulations (Fig. 4I through L). After 1 µs of atomistic simulation, visual inspection revealed that all peptides remained stably embedded within the membrane, and the peptide aggregates were maintained, as shown in the lower panels of Fig. 4. While these initial observations provide valuable insights into the stability and persistence of peptide aggregates, they do not fully elucidate the impact of these oligomeric structures on membrane properties. To address this, we next evaluated the membrane-perturbing effects induced by the proposed FPs.

### iFP and nFP exhibit different membrane-perturbing effects in MD simulations

**Fig 5:**
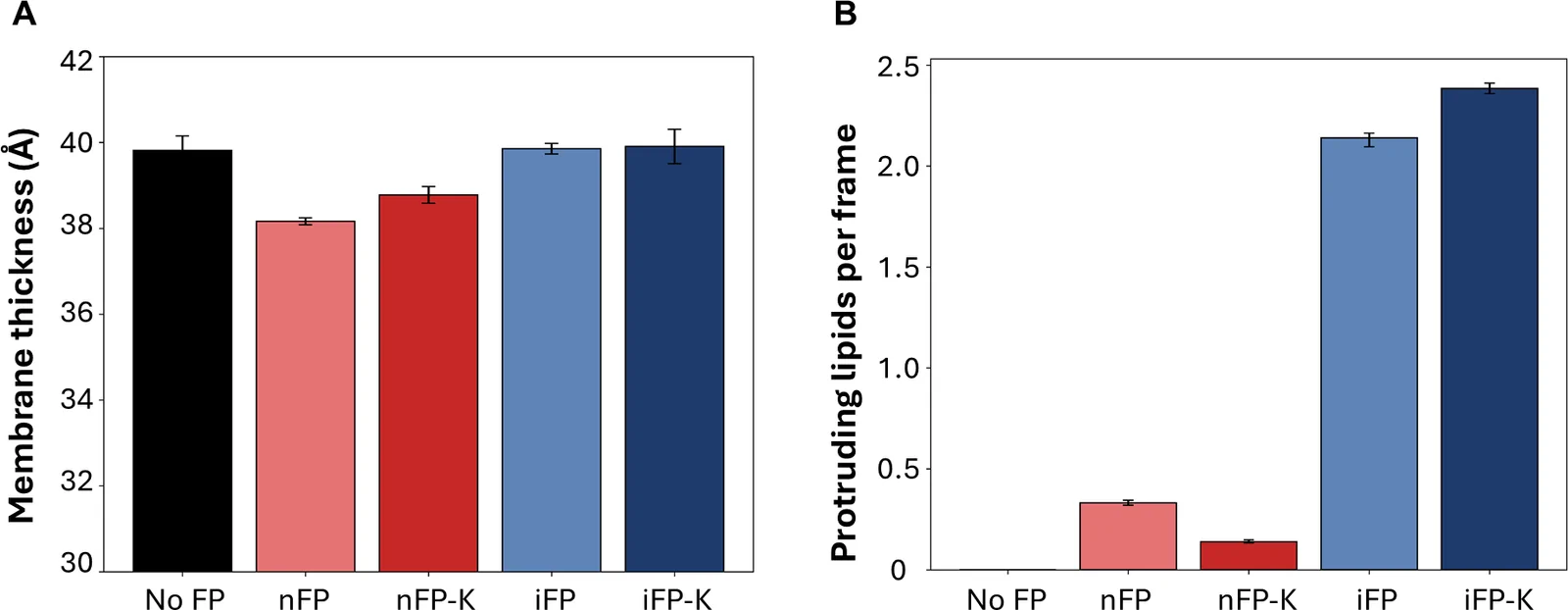
Effects of proposed SARS-CoV-2 FPs on membrane properties analyzed *in silico*. (**A**) Membrane thickness and (**B**) number of protruding lipids per frame determined over the last 500 ns of three independent atomistic MD simulation replicas for systems containing no peptide (black), nFP (pink), nFP-K (red), iFP (light blue), and iFP-K (dark blue). Error bars were calculated with bootstrap resampling and correspond to the 95% confidence intervals.

To explore the structural consequences of the proposed SARS-CoV-2 FPs’ presence and aggregation within the membrane, we evaluated membrane thickness as an indicator of perturbation. Changes in bilayer thickness can reflect alterations in lipid packing and local deformation, particularly in response to embedded peptides. Our analysis revealed that the presence of nFP and nFP-K led to a thinning of the membrane (Fig. 5A). This thinning effect is consistent with the wedge-shaped conformation of nFP, which is thought to promote local distortion of the bilayer architecture. In contrast, iFP and iFP-K exerted minimal influence on membrane thickness, with only slight variations observed.

To further evaluate the membrane-perturbing effects of the FPs, we analyzed one of the computational hallmarks of membrane perturbation: lipid tail protrusion (75–77). This phenomenon, first identified in MD simulations, is defined by the outward extension of lipid tails beyond their corresponding phosphate headgroups. To quantify this effect, we measured the number of lipid tail protrusion events occurring within the last 500 ns of the atomistic MD simulations. Our analysis revealed that both FPs proposed induce lipid tail protrusions, but to considerably different extents. Specifically, iFP and iFP-K triggered approximately five times as many protrusion events compared to nFP and nFP-K, indicating a greater capacity of iFP for membrane perturbation (Fig. 5B). These findings suggest that while both proposed FPs perturb the membrane, the mechanisms by which they do so may differ: nFP variants appear to deform the bilayer more through lateral stress and thinning, whereas iFP variants induce perturbation through deeper insertion and increased lipid dynamics, such as lipid tail protrusion.

### iFP oligomeric assemblies allow water flux through the membrane

**Fig 6:**
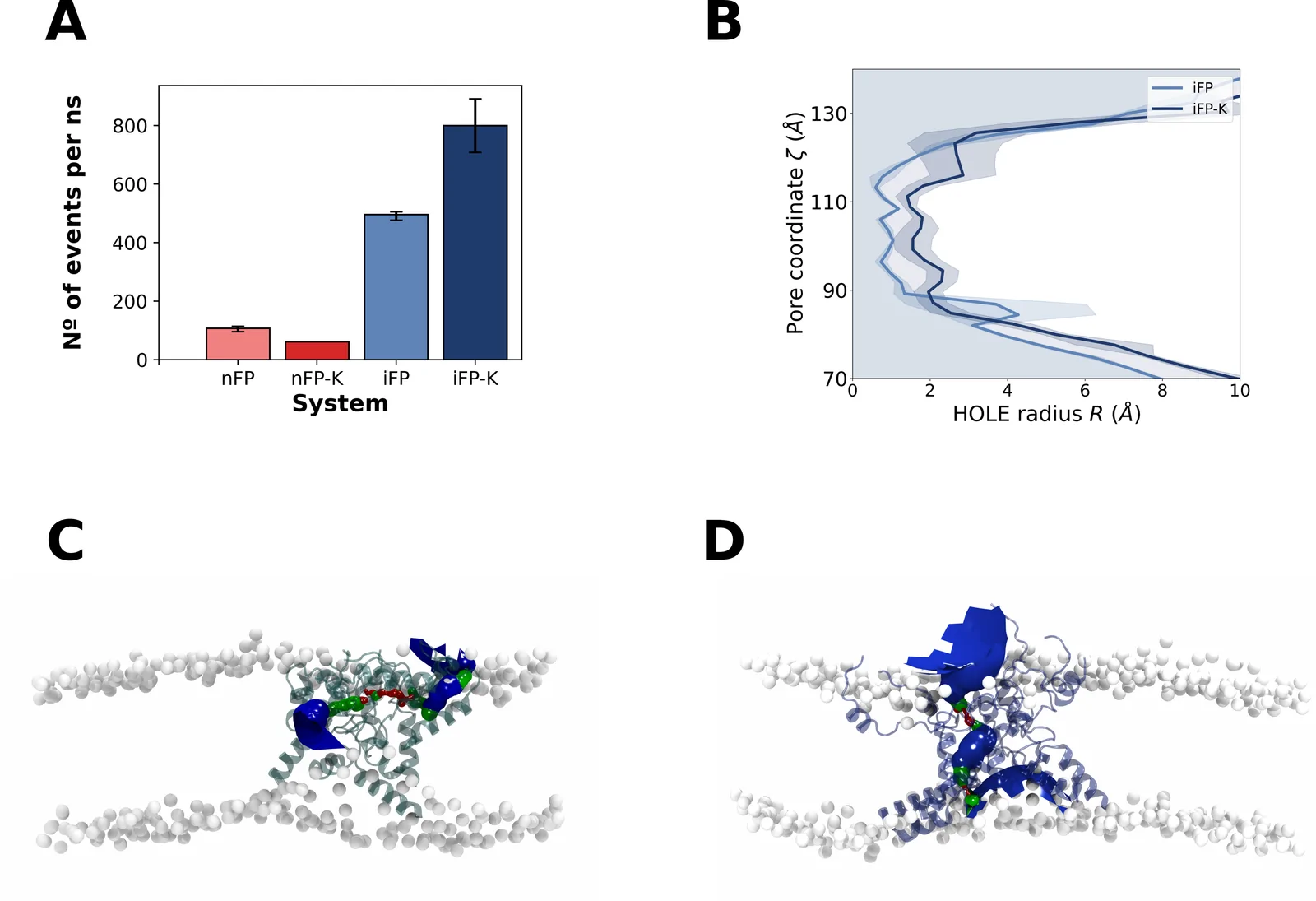
Characterization of the pore formed within the oligomeric assemblies of proposed SARS-CoV-2 FPs. (**A**) Number of water-crossing events through the membrane for the systems containing nFP (pink), nFP-K (red), iFP (light blue), and iFP-K (dark blue). Error bars were calculated with bootstrap resampling and correspond to the 95% confidence intervals. (**B**) Radius of the pores formed by the oligomeric structures of iFP (light blue) and iFP-K (dark blue). The pore radius was determined using the spherical probe of the HOLE algorithm (61). (**C and D**) Representative snapshots generated with the HOLE2 analysis tool from MD simulation analyses that show the accessibility of water into the membrane for the iFP and iFP-K systems, respectively.

To gain molecular-level insight into how oligomeric structures formed by the FPs affect membrane permeability, we investigated water flux in the *in silico* systems. Motivated by the experimentally observed vesicle content leakage, we analyzed the space within the oligomeric structures formed by the proposed SARS-CoV-2 FPs and quantified the number of water molecules passing through these structures, aiming to characterize the molecular pathways that enable water permeation. This analysis was performed on the atomistic MD simulations using the HOLE method (60, 61) combined with the fluxer.py script. Water flux through the membrane was more pronounced in the presence of either iFP or iFP-K, whereas nFP and nFP-K contributed much less to water permeation (Fig. 6A). Interestingly, both iFP and iFP-K formed pore-like structures with constriction radii of approximately 1.5 Å and 2 Å, respectively (Fig. 6B)—both exceeding the 1.15 Å radius required to accommodate a water molecule. Our results indicate that even in the narrowest regions of these oligomeric structures, water molecules can pass through along their entire length. Notably, pores formed by iFP were generally narrower than those formed by iFP-K (Fig. 6C and D).

The computational findings regarding membrane perturbation and water flux through the membrane align with the experimental data, in which iFP demonstrated a more pronounced ability to induce both phenomena. Interestingly, in our simulations, the effects of iFP and iFP-K were comparable, in contrast to experimental observations where the poly-lysine tail was shown to significantly enhance membrane interaction. This discrepancy likely arises from the simulation setup, where peptides were pre-inserted into the membrane, thereby bypassing the solubility and membrane-partitioning barriers present in *in vitro* conditions. Previous studies on the parainfluenza fusion peptide from a class I fusion protein reported similar behavior, further supporting the idea of a conserved mechanism by which class I fusion peptides promote membrane fusion (19, 20).

While the membrane-level analyses provide valuable insights into the overall perturbation effects of the proposed FPs, they do not fully capture the underlying molecular mechanisms driving these phenomena. To gain a deeper understanding of how these peptides interact with the membrane and with each other, we next investigated residue-level interactions and molecular contacts within the systems.

### F817 and the LLF motif of nFP contribute to membrane anchoring and bilayer destabilization

**Fig 7:**
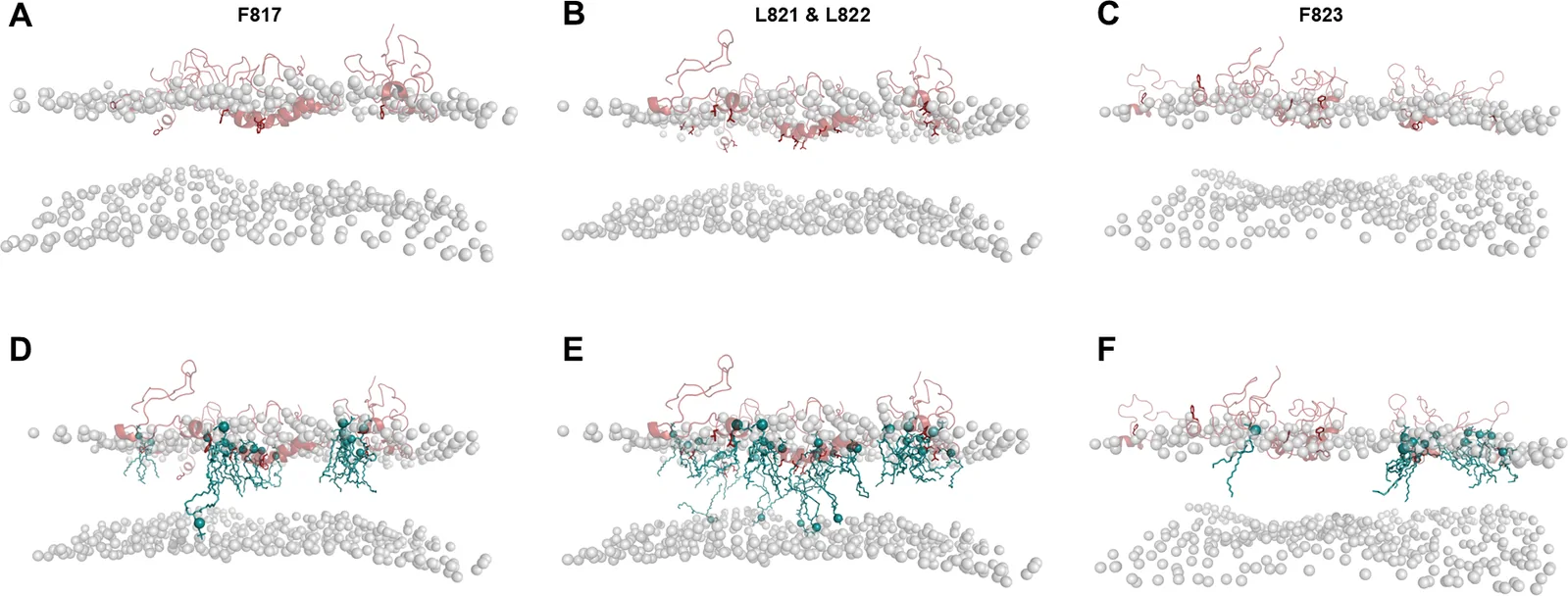
Structural representation of key nFP residue-lipid interactions. Panels **A–C** show the membrane-inserted peptide highlighting the position of residues F817 (**A**), L821 and L822 (**B**), and F823 (**C**). Peptide copies are depicted as red cartoons, with the side chains of the residues of interest shown as sticks. Only the phosphate headgroups of the lipid bilayer are displayed as gray spheres for visual clarity purposes. Panels **D–F** display the phospholipids interacting with F817 (**D**), L821/L822 (**E**), and F823 (**F**). In these panels, interacting lipids are shown as cyan sticks, with their phosphate groups highlighted as spheres. Lipids from both leaflets are included, illustrating the residue-specific lipid interactions across the membrane.

The N-terminal regions of nFP and nFP-K are located in the membrane at the level of the phosphate headgroups. This peptide region, composed of the SFIEDLLF sequence, tends to form an alpha-helix when associated with the membrane interface. A key residue, F817, consistently orients inward toward the membrane core (Fig. 7A), where it interacts directly with the lipid acyl chains, effectively anchoring the helix. Remarkably, F817 occupies a position within the membrane deep enough to contact lipid tails from both membrane leaflets, suggesting a mechanism that promotes bilayer destabilization and possibly contributes to membrane thinning. This thinning may occur through pulling together phospholipids from opposing leaflets, bringing them into closer proximity (Fig. 7D). Although F817 appears to play an important role in SARS-CoV-2 nFP membrane insertion, it tolerates conservative substitutions to alanine or leucine across beta-coronaviruses (Fig. 1C). These substitutions generally reduce, but do not abolish, viral entry (24, 34). Collectively, these findings suggest that while F817 optimizes peptide anchoring and positioning within the membrane, non-aromatic hydrophobic residues can still support sufficient partitioning, thereby preserving function and allowing a degree of evolutionary flexibility.

In addition to F817, the LLF motif (residues L821–F823) also plays a crucial role in membrane anchoring. Both leucine residues adopt an inward-facing orientation, their side chains interacting with lipid tails from both leaflets, similarly to F817 (Fig. 7B and E). These interactions likely contribute to bilayer destabilization and membrane narrowing in the regions where the peptides are located. F823 exhibits a different behavior: it alternates between facing inward and outward relative to the membrane but primarily interacts with the headgroups of phospholipids (Fig. 7C and F). This shallower position contrasts with the deep membrane-anchoring roles of F817. The LLF motif is highly conserved among coronaviruses, and mutagenesis studies targeting this motif have shown that fusion activity is significantly reduced or completely abolished when any of these residues are substituted. Despite expressing spike in the correct prefusion conformation, pseudoviruses carrying such mutations exhibit severely impaired infectivity, underscoring the functional importance of this motif in membrane fusion (34).

**Fig 8:**
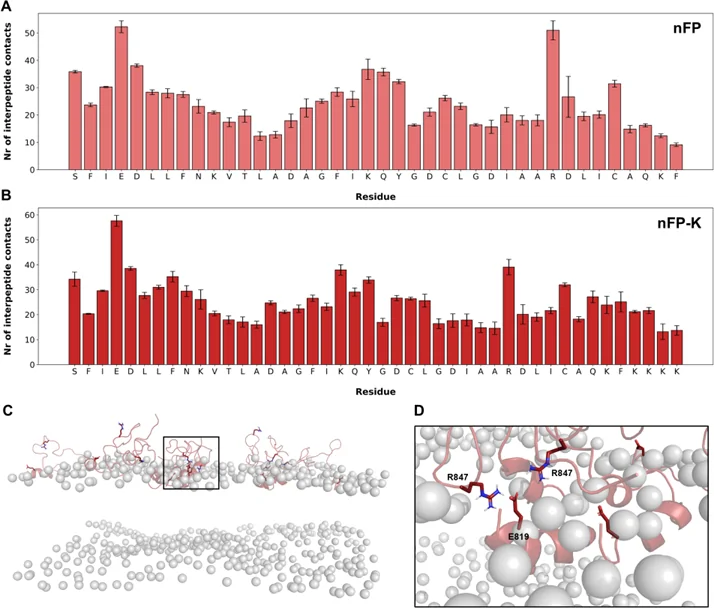
Interpeptide interactions in nFP and nFP-K. Panels **A and B** display the number of interpeptide contacts formed by each residue of the nFP (**A**) and nFP-K (**B**) peptides, identifying regions involved in frequent interactions. Error bars were calculated with bootstrap resampling and correspond to the 95% confidence intervals. In panel **C**, the positions and orientations of residues E819 and R847 from multiple peptides are shown to illustrate their spatial arrangement and potential for interpeptide interaction. Peptides are represented as red cartoons, with E819 and R847 side chains depicted as sticks. Panel **D** shows a close-up view of the interaction interface between E819 and R847, highlighting their side chain conformations and proximity. For visualization purposes, the membrane is represented only by the phosphate groups of both leaflets, shown as gray spheres.

Small clusters of 2–4 peptides appear to form through interpeptide interactions, particularly involving E819 and R847 (Fig. 8A and B). While R847 is typically solvent-exposed and exhibits high conformational flexibility, E819 is more stably positioned at the membrane interface, where it localizes near the phosphate headgroups. The remaining sequence adopts a predominantly disordered conformation outside the membrane, exhibiting dynamic fluctuations between more compact states near the membrane interface and more extended conformations toward the solvent (Fig. 8C and D). E819 and R847 residues, along with three conserved aspartic acid residues in the sequence, are also highly conserved across coronaviruses and thought to play roles in ion coordination or binding, potentially modulating membrane curvature or fusion dynamics (30, 78–80).

### iFP residues mediate a membrane pulling mechanism and stabilize oligomeric assemblies

**Fig 9:**
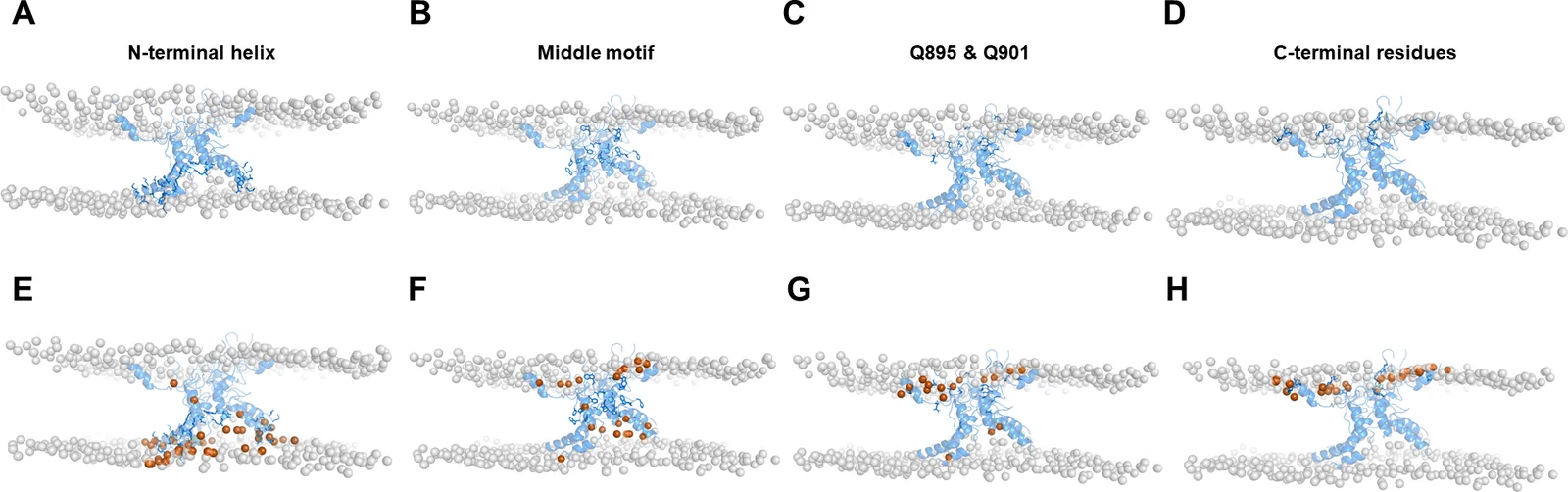
Structural representation of key iFP residue-lipid interactions. Panels **A–D** show the membrane-inserted peptide highlighting the position of the N-terminal helix residues (**A**), middle motif residues (**B**), Q895 and Q901 (**C**), and the C-terminal residues (**D**). Peptide copies are depicted as blue cartoons, with the side chains of the residues of interest shown as sticks. Only the phosphate headgroups of the lipid bilayer are displayed as gray spheres for visual clarity purposes. Panels **E–H** display the phospholipids interacting with the N-terminal helix residues (**E**), middle motif residues (**F**), Q895 and Q901 (**G**), and C-terminal residues (**H**). In these panels, interacting headgroup lipids are shown as orange spheres. Lipids from both leaflets are included, illustrating the residue-specific lipid interactions across the membrane.

In contrast to the nFP, both iFP and iFP-K are fully embedded within the lipid bilayer and form stable oligomeric aggregates in the membrane. Specific segments of the iFP engage in frequent interactions with the phospholipid headgroups, which contribute both to its stable positioning within the membrane and to its membrane-perturbing activity. These membrane-interacting regions can be categorized into four main elements: an N-terminal helix, a central motif, a pair of glutamine residues (Q895 and Q901), and the two C-terminal residues (Fig. 9A through D).

The N-terminal helix (residues 869–873, sequence MIAQY) primarily interacts with the phospholipid headgroups of the lower leaflet. This interaction induces a localized inward protrusion of the lower leaflet, as the helical segment is located deeper into the bilayer. This likely reflects a “pulling” mechanism, in which the peptide draws the inner leaflet upward, contributing to membrane curvature and asymmetry (Fig. 9E). Similarly, the middle motif (residues 886–888, sequence WTF) engages with lipid headgroups but interacts distinctively with both leaflets of the bilayer. This dual interaction appears to bridge the polar headgroups across the membrane midplane, promoting deeper headgroup intrusion into the hydrophobic core. Such behavior may enhance local bilayer thinning and facilitate membrane destabilization, conditions that are favorable for membrane fusion events (Fig. 9F).

The two glutamine residues (Q895 and Q901) predominantly interact with headgroups from the upper leaflet (Fig. 9G). Along with the two C-terminal residues (Y904 and R905), these appear to act as a membrane anchor, stabilizing the orientation of the peptide within the bilayer (Fig. 9H). The polar side chains of glutamines form hydrogen bonds with phosphate or carbonyl groups of the lipids, reinforcing the peptide’s embedded conformation. These residues are largely conserved across beta-coronaviruses, reflecting their role in membrane interaction. However, each position can tolerate mutation to polar or small nonpolar residues. This tolerance likely arises because polar residues might partially mimic glutamine’s hydrogen-bonding interactions, while small nonpolar residues could be accommodated without steric clashes, allowing the peptide to maintain overall membrane anchoring and orientation. The role of glutamine residues within fusion peptides of class I viral fusion proteins has been demonstrated previously in the parainfluenza fusion peptide, where substitution of glutamine residues led to altered peptide oligomerization within the membrane and reduced water flux across the membrane (20).

**Fig 10:**
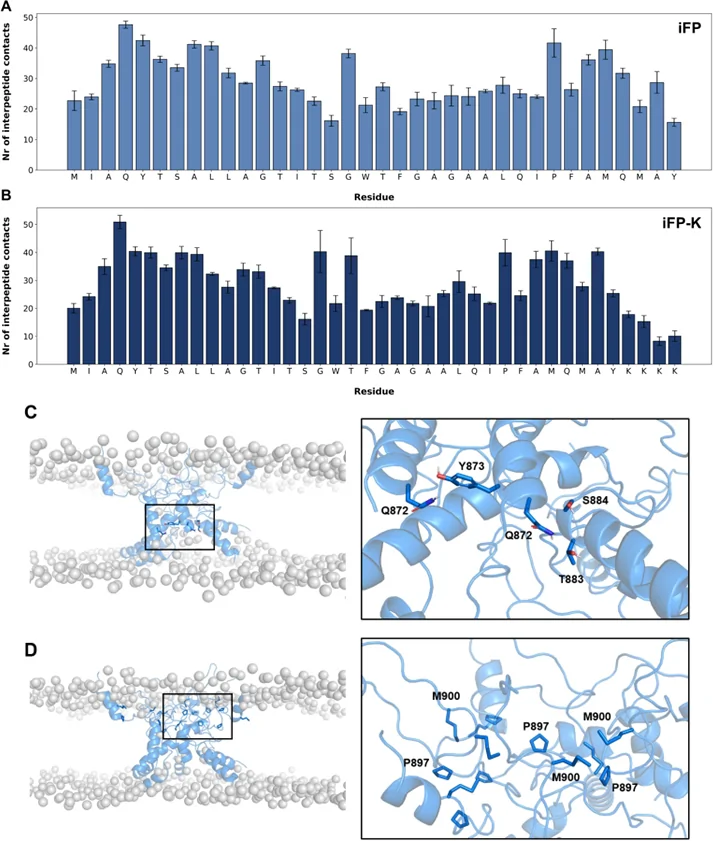
Interpeptide interactions in iFP and iFP-K. Panels **A and B** display the number of interpeptide contacts formed by each residue of the iFP (**A**) and iFP-K (**B**) peptides, identifying regions involved in frequent interactions. Error bars were calculated with bootstrap resampling and correspond to the 95% confidence intervals. In panels **C and D**, the positions of residues Q872, Y873, T883, and S884 (**C**) and P897 and M900 (**D**) from multiple peptides are shown to illustrate their spatial arrangement and potential for interpeptide interaction. Peptides are represented as blue cartoons, with residues of interest side chains depicted as sticks. For visualization purposes, the membrane is represented only by the phosphate groups of both leaflets, shown as gray spheres.

The oligomeric aggregates formed by iFP and iFP-K peptides are stabilized through a combination of interpeptide interactions, involving several amino acid residues (Fig. 10A and B). For example, Q872 participates in intermolecular hydrogen bonding with neighboring residues such as Y873, T883, and S884, forming a network of polar contacts that help stabilize the assembly (Fig. 10C). These interactions likely support peptide alignment and partial ordering within the aggregate. Specific interpeptide interactions, particularly involving glutamine and threonine residues, have been identified as essential for proper oligomerization of class I fusion peptides within the membrane and for their membrane-perturbing activity (19, 20).

In more disordered regions of the peptide, P897 and M900 contribute to the formation of localized hydrophobic clusters at interpeptide interfaces. These residues facilitate peptide-peptide association through non-polar interactions, despite the lack of regular secondary structure in these segments (Fig. 10D). In contrast, alanines and leucines tend to mediate hydrophobic interactions in regions adopting alpha-helical conformations. Their side chains, when presented on the same helical face, may facilitate helix-helix packing and promote a more ordered oligomeric architecture. Within these intrinsically disordered segments, conformational flexibility also plays an important role in oligomer stabilization. G885, located in these regions, contributes significantly to their dynamic character due to the minimal steric constraints of glycine, allowing transient contacts with residues from adjacent peptides. Such flexibility may facilitate local rearrangements and adaptability within the oligomeric structure (18).

### Conclusions

In this work, we combined biophysical and computational approaches to elucidate the membrane-interacting properties of two proposed SARS-CoV-2 FPs: nFP and iFP. These two peptides were analyzed without any modification to their sequence or with a C-terminal poly-lysine tail to improve solubility. Our *in vitro* data indicate that nFP and nFP-K bind weakly to the membrane, and the MD simulations suggest that they induce moderate membrane thinning, consistent with a potential role in priming the membrane for fusion. The N-terminal F817 residue acts as an anchor point, while the conserved LLF motif is more deeply localized in the membrane and promotes cross-leaflet lipid interactions that may locally destabilize the bilayer and facilitate subsequent membrane fusion steps. In contrast, our *in vitro* biophysical analyses show that the iFP-K displayed higher membrane affinity and induced more pronounced membrane perturbations, including content flux through the membrane. The simulations indicate that distinct iFP and iFP-K regions interact with phospholipids from both membrane leaflets, generating a “membrane pulling” effect that could contribute to membrane fusion. Moreover, pseudoviruses carrying mutations in iFP aromatic residues exhibited markedly reduced infectivity, although the S-protein retained a functional prefusion conformation, underscoring the crucial role of these residues—and the iFP as a whole—in efficient viral entry.

**Fig 11:**
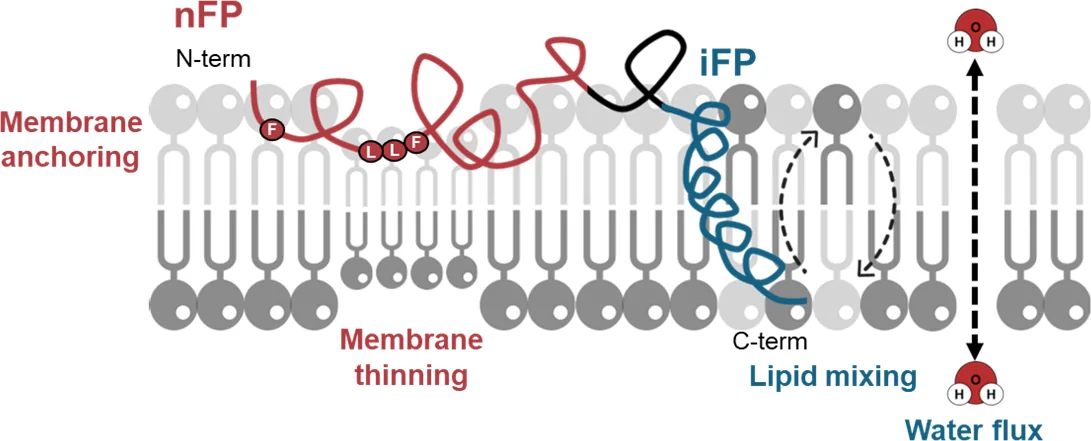
Proposed model for the mechanism of action of the SARS-CoV-2 spike protein bipartite fusion module. The scheme summarizes the main findings of this study and illustrates the distinct roles of each fusion peptide during viral entry. The N-terminal fusion peptide (nFP) is shown in red, with its key membrane interactions and associated effects highlighted accordingly. The internal fusion peptide (iFP) is shown in blue, together with its corresponding membrane-perturbing effects.

Recent cryo-EM studies of the SARS-CoV-2 S-protein in both the S2′-cleaved and postfusion states, reconstituted in nanodiscs, revealed that residues 816–834 are positioned shallowly within, or just beneath, the lipid headgroup region, whereas residues 835–856 localize at the membrane interface and interact closely with the iFP (26, 81). Complementing these structural observations, recent CG MD simulations showed that the nFP region (residues 816–856) forms membrane-associated aggregates capable of inducing convex membrane curvature, whereas part of the iFP (residues 889–906) assembles into aggregates that promote membrane pore formation (82). Together, these findings suggest that the nFP functions primarily as a fusion-supportive structural element that facilitates membrane remodeling, while the iFP penetrates deeper into the membrane and plays a more direct role in destabilizing the membrane. Overall, these complementary structural and computational observations further support our hypothesis that the nFP and iFP perform distinct yet complementary functions during the membrane fusion process. We propose that the nFP functions at an earlier stage of fusion, initiating membrane engagement, facilitating iFP deeper insertion in the membrane, and consequent membrane destabilization driving membrane fusion, as depicted in Fig. 11. Ultimately, the action of both peptides might lead to the transition to the stabilized postfusion S-protein conformation.

Overall, this study advances our understanding of the molecular mechanisms underlying SARS-CoV-2 S-protein-mediated membrane fusion and viral entry. Given that the S-protein fusion machinery is highly conserved across coronaviruses, the mechanism here uncovered may represent a general feature of coronavirus FPs, providing broader insight into how this viral family achieves membrane fusion. These findings may also aid the development of antiviral strategies targeting this highly conserved and functionally crucial region of the S-protein.

## Data Availability

The data supporting this article are available within the article and in Zenodo at https://doi.org/10.5281/zenodo.22033057. The raw files are available upon request.

## SUPPLEMENTAL MATERIAL

Supplemental material jvi.00688-26-s0001.pdf Table S1; Fig. S1 and S2.

ASM does not own the copyrights to Supplemental Material that may be linked to, or accessed through, an article. The authors have granted ASM a non-exclusive, world-wide license to publish the Supplemental Material files. Please contact the corresponding author directly for reuse.
