# Supplementary material for: Insights into the mechanism of action of the bipartite fusion module of SARS-CoV-2 spike protein
Source: J Virol. 2026 Sep 15;100(10):e00688-26. doi: 10.1128/jvi.00688-26 (PMC13712820; doi:10.1128/jvi.00688-26)
Supplement: Supplementary file 1 [file jvi.00688-26-s0001.pdf]

# Insights into the mechanism of action of the bipartite fusion module of SARS-CoV-2 spike protein

Carolina C. Buga[1,2,3], Mariana Valério[1], Marta Alenquer[4], Marta Pires de Miranda[2,3,4], Manuel N. Melo[1], Miguel A. R. B. Castanho[2,3], Maria João Amorim[4], Cláudio M. Soares[1], João B. Vicente[1*], Ana Salomé Veiga[2,3*], Diana Lousa[1*]

[1]Instituto de Tecnologia Química e Biológica António Xavier, Universidade Nova de Lisboa, Oeiras, Portugal

[2]Faculdade de Medicina, Universidade de Lisboa, Lisboa, Portugal

[3]Gulbenkian Institute for Molecular Medicine, Lisboa, Portugal

[4]Católica Biomedical Research Centre, Católica Medical School – Universidade Católica Portuguesa, Oeiras, Portugal

[*]Corresponding authors

## Supplemental material

**Table S1 |** Spike-pseudotyped lentiviruses with mutations in the iFP region. Each mutant number (Mut #) corresponds to a pseudovirus construct carrying a single or double amino acid mutations within the iFP region of the SARS-CoV-2 S-protein.

| Mut # | Mutation in the iFP region |
|---|---|
| Mut1 (Y873A) | MIAQ**A**TSALLAGTITSGWTFGAGAALQIPFAMQMAYR(F) |
| Mut2 (W886A) | MIAQYTSALLAGTITSG**A**TFGAGAALQIPFAMQMAYR(F) |
| Mut3 (F888A) | MIAQYTSALLAGTITSGWT**A**GAGAALQIPFAMQMAYR(F) |
| Mut4 (F898A) | MIAQYTSALLAGTITSGWTFGAGAALQIP**A**AMQMAYR(F) |
| Mut5 (Y904A) | MIAQYTSALLAGTITSGWTFGAGAALQIPFAMQMA**A**R(F) |
| Mut6 (F906A) | MIAQYTSALLAGTITSGWTFGAGAALQIPFAMQMAYR(**A**) |
| Mut7 (W886A/F888A) | MIAQYTSALLAGTITSG**A**T**A**GAGAALQIPFAMQMAYR(F) |
| Mut8 (F898A/F906A) | MIAQYTSALLAGTITSGWTFGAGAALQIP**A**AMQMAYR(**A**) |
| Mut9 (Y873A/Y904A) | MIAQ**A**TSALLAGTITSGWTFGAGAALQIPFAMQMA**A**R(F) |
| Mut10 (F898Y/F906Y) | MIAQYTSALLAGTITSGWTFGAGAALQIP**Y**AMQMAYR(**Y**) |
| Mut11 (F898W/F906W) | MIAQYTSALLAGTITSGWTFGAGAALQIP**W**AMQMAYR(**W**) |
| Mut12 (Y873F/Y904F) | MIAQ**F**TSALLAGTITSGWTFGAGAALQIPFAMQMA**F**R(F) |
| Mut13 (Y873W/Y904W) | MIAQ**W**TSALLAGTITSGWTFGAGAALQIPFAMQMA**W**R(F) |
| Mut14 (W886Y) | MIAQYTSALLAGTITSG**Y**TFGAGAALQIPFAMQMAYR(F) |
| Mut15 (W886F) | MIAQYTSALLAGTITSG**F**TFGAGAALQIPFAMQMAYR(F) |

To confirm that all pseudoviruses effectively displayed the S-protein, western blot was performed on the viral particle suspensions. The majority of pseudoviruses blots probed with an anti-S1 antibody consistently showed two bands: one at approximately 100 kDa, corresponding to the cleaved and glycosylated S1 subunit derived from the S-protein, and another above 75 kDa (Figure S1). The latter likely represents a non-specific signal from host or serum proteins incorporated into the viral preparation, as similar cross-reactivity has been reported in pseudovirus western blot analyses [1-3]. Although the full-length non-cleaved S-protein (band near 180 kDa) was detected as a positive control, it was not visible in the pseudovirus-derived samples, consistently with previous reports that furin-mediated S1/S2 cleavage and subsequent S1 shedding during pseudovirus production and purification result in virions predominantly bearing the cleaved S-protein form [1, 2].

S-protein incorporation occurred across most pseudoviruses mutants; however, pseudoviruses carrying F898 mutations lacked detectable S-protein (Figure S1), indicating a potential defect in S-protein incorporation. This finding suggests that residue F898 might play a key structural role in maintaining S-protein stability or its incorporation into the pseudoviral particle. To further investigate this, structural predictions of the F898 substituted S-proteins were generated using AlphaFold3 (AF3) [4] (Figure S2). The models showed reduced confidence in the following regions: N-terminal domain (NTD) (residues 68-264), S1/S2 interface (residues 620-640), S1/S2 cleavage site region (residues 677-688), and part of nFP (residues 827-855) compared to the SARS-CoV-2 WT S-protein structure. These regions correspond to functionally essential regions involved in antigenicity (NTD) [5], proteolytic activation (S1/S2 cleavage site) [6], and membrane fusion (nFP) [7]. Thus, perturbations in their conformation could have implications for viral infectivity or immune evasion. Among these, the nFP region (827–855) exhibited the largest decrease in confidence scores and increased structural variability. This suggests that the F898 mutation, although located outside this segment, may induce structural or allosteric rearrangements that disrupt the local organization or interactions essential for maintaining the correct conformation of the fusion machinery. Given the importance of proper trimeric S-protein folding and stability for efficient trafficking and incorporation into budding pseudovirus particles, such destabilization is likely to contribute to the observed defect in S-protein incorporation in pseudovirus particles.

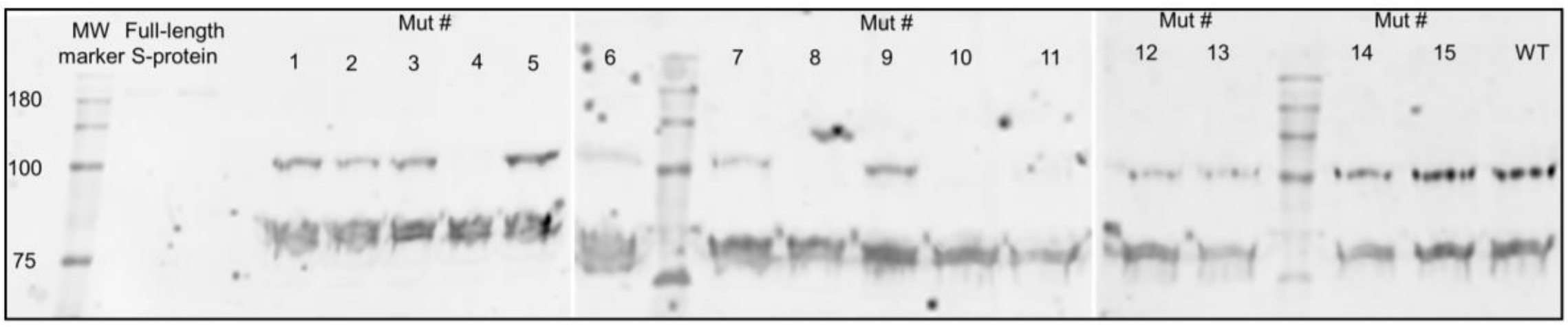


**Figure S1 |** Western blot analysis of spike-pseudotyped lentiviral particles, confirming the expression and incorporation of WT and S-protein mutants in pseudoviral particles. The WT corresponds to the SARS-CoV-2 D614G (B.1 lineage) S-protein, used as a control for functional assays and to validate proper S-protein incorporation into pseudoviral particles. Full-length S-protein was used as a control to verify antibody specificity, ensuring accurate detection of S-protein across samples.

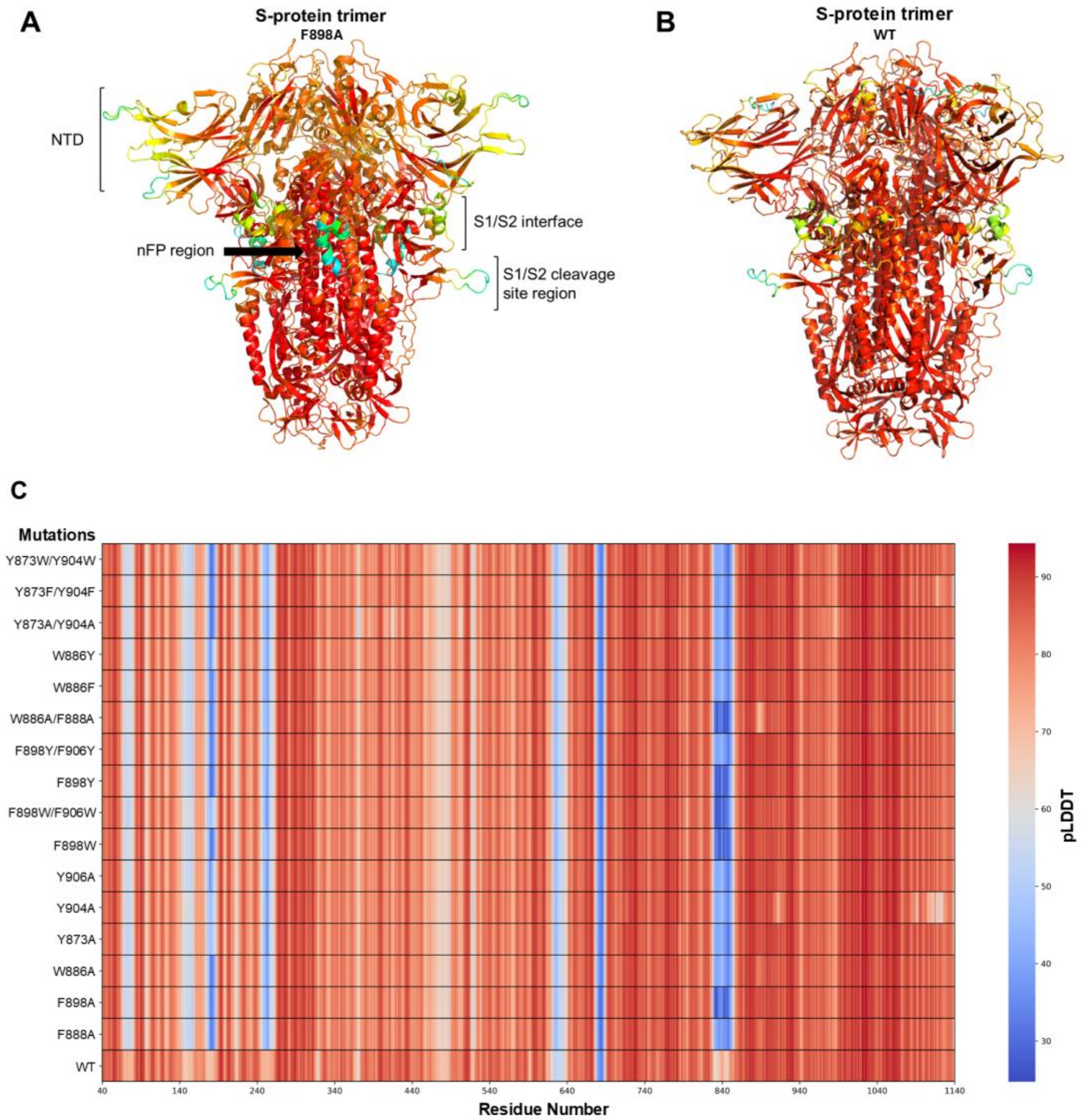


**Figure S2 |** Structural predictions of substituted SARS-CoV-2 S-proteins generated using AlphaFold3 (AF3). Predicted three-dimensional structures of the (A) F898A and (B) wild-type (WT) S-proteins generated using AF3. Structural confidence is depicted using a color gradient, where red indicates regions with high prediction confidence and blue denotes regions with low confidence, based on the predicted Local Distance Difference Test (pLDDT) scores. The pLDDT score is a per-residue metric ranging from 0 to 100, with higher values reflecting greater confidence in the predicted local atomic positions. (C) Comparison of pLDDT score profiles across substituted S-proteins. Functionally and structurally important regions are highlighted, including the N-terminal domain (NTD), the S1/S2 interface, the S1/S2 cleavage site region, and part of the N-terminal fusion peptide (nFP).

**References:**

[1] Zhang L, Hom N, Ojha A, Lovendahl KN, Mou H, Lee KK, Choe H. Cytoplasmic tail truncation stabilizes S1-S2 association and enhances S protein incorporation into SARS-CoV-2 pseudovirions. J Virol. 2023;97(3):e01650-22. doi:10.1128/jvi.01650-22.

[2] Wang Q, Nair MS, Anang S, Zhang S, Nguyen H, Huang Y, Liu L, Ho DD, Sodroski JG. Functional differences among the spike glycoproteins of multiple emerging severe acute respiratory syndrome coronavirus 2 variants of concern. iScience. 2021;24(11):103393. doi:10.1016/j.isci.2021.103393.

[3] Li Y, Zhang X, Tai W, Zhuang X, Shi H, Liao S, Yu X, Mei R, Chen X, Huang Y, Liu Y, Liu J, Liu Y, Zhu Y, Wang P, Tian M, Yu G, Li L, Cheng G. A substitution at the cytoplasmic tail of the spike protein enhances SARS-CoV-2 infectivity and immunogenicity. EBioMedicine. 2024;110:105437. doi:10.1016/j.ebiom.2024.105437.

[4] Abramson J, Adler J, Dunger J, et al. Accurate structure prediction of biomolecular interactions with AlphaFold 3. Nature. 2024;630:493-500. doi:10.1038/s41586-024-07487-w.

[5] Ciccozzi M, Pascarella S. Two sides of the same coin: the N-terminal and the receptor binding domains of SARS-CoV-2 spike. Future Virol. 2023;18:2217-fvl-2022-0181. doi:10.2217/fvl-2022-0181.

[6] Xia X. Domains and functions of spike protein in SARS-CoV-2 in the context of vaccine design. Viruses. 2021;13(1):109. doi:10.3390/v13010109.

[7] Li X, Yuan H, Li X, Wang H. Spike protein mediated membrane fusion during SARS-CoV-2 infection. J Med Virol. 2023;95(1):e28212. doi:10.1002/jmv.28212.
